# Utilization of structural steel in buildings

**DOI:** 10.1098/rspa.2014.0170

**Published:** 2014-08-08

**Authors:** Muiris C. Moynihan, Julian M. Allwood

**Affiliations:** Department of Engineering, University of Cambridge, Trumpington Street, Cambridge CB2 1PZ, UK

**Keywords:** steel, material efficiency, construction, structural design, building design, rationalization

## Abstract

Over one-quarter of steel produced annually is used in the construction of buildings. Making this steel causes carbon dioxide emissions, which climate change experts recommend be reduced by half in the next 37 years. One option to achieve this is to design and build more efficiently, still delivering the same service from buildings but using less steel to do so. To estimate how much steel could be saved from this option, 23 steel-framed building designs are studied, sourced from leading UK engineering firms. The utilization of each beam is found and buildings are analysed to find patterns. The results for over 10 000 beams show that average utilization is below 50% of their capacity. The primary reason for this low value is ‘rationalization’—providing extra material to reduce labour costs. By designing for minimum material rather than minimum cost, steel use in buildings could be drastically reduced, leading to an equivalent reduction in ‘embodied’ carbon emissions.

## 1. Introduction: the need to use our resources efficiently

Every year over 1500 million tonnes of steel are produced [1], causing 9% of global carbon dioxide emissions from energy and processes [2]. Allwood *et al.* [3] forecast that demand for steel will double in the next 37 years, and Müller *et al.* [4] show that iron ore reserves will not exhaust in this period (although increasingly inaccessible ore deposits may become economically and technically challenging to exploit). However, climate change experts recommend that emissions are halved by 2050 to avert the worst repercussions of global warming [5]. Coupled with rising demand, achieving this target will necessitate a 75% reduction in emissions per kilogram of steel produced, but Allwood *et al.* [3] report that insufficient efficiency improvements remain in steel-making processes to do so. Emissions could possibly be reduced through increased use of renewable energy or carbon capture and storage networks, but Smil [6] lists the formidable logistical obstacles to implementing these technologies at a commensurate scale in the timeframe. Another option to reduce emissions is material efficiency—delivering the same final services with reduced new production. Allwood *et al.* [7] list six material efficiency strategies, of which ‘using less metal by design’ is one. Half of steel is used by the construction industry [8], of which 60% is used in buildings [9], so using less steel in buildings would significantly reduce carbon emissions.

## 2. Review of previously published literature

Austin [10, p. 17] states that ‘the prime objective of an engineer [is] to produce a robust solution … in the most economic and practical way possible’. Determining the potential to reduce the steel in buildings therefore requires an understanding of both structural design and construction economics. Structural design guidance is reviewed in §2*a* to chart the advances in engineering knowledge over the past century and the subsequent changes in material requirements to provide a safe structure. Guidance on designing structures economically is reviewed over this period in §2*b*, examining the evolution of labour costs relative to those of materials and how this impacts on achieving an economic structural design.

### (a) History of structural steel design guidance

As human understanding of structural steel's material properties and behaviour has improved, it has become possible to design safely for the same applied load with less and less material. Bates [11] describes the history of iron and steel in construction, listing the improvements in steel production that resulted in more consistent material properties. Production improvements have been so successful that Eurocodes, described by Nethercot [12] as ‘technically the most advanced’ design standards, allow engineers to assume full material strength when designing structural steel [13]. Beal [14] builds on Bates' chronology to chart the reduction of ‘safety margins’ (allowances in design for uncertainty in material property, behaviour or loading) in UK design standards from a factor of 4—when steel sections were first produced in quantity for construction in the 1880s—to 1.3–1.45 for Eurocode 3, which superseded previous design codes in 2010. Beal notes that advances in engineering theory—such as the introduction of plasticity theory in 1959—as well as material property improvements allowed more of steel's strength to be exploited. Continuation of this trend is borne out in recent comparisons between the British Standards and the Eurocodes which are replacing them: Webster [15] finds that Eurocode uses 2% less material for a concrete framed building, while Moss & Webster [16] conclude that Eurocode ‘offers scope for more economic structures’.

Despite a century of progress, there is further scope for improvement of design standards as highlighted by studies such as that of Hicks [17], who compares experimental results with predictions from Eurocode, finding strengths almost double those predicted in a majority of cases. This is affirmed by other studies which show ways for even less material to be used: Carruth *et al.* [18] demonstrate that an optimized, varying cross-section beam could have 30% less mass than a standard beam; Chan's [19] optimization method reduces steel mass of a 60-storey building by 3.5%, while Liang *et al.* [20] presents a performance-based topology optimization method to minimize the mass of structural bracing systems.

### (b) Review of strategies to reduce building cost

At the beginning of the twentieth century, when steel was becoming widely used in construction, BCSA [21] notes that labour was comparatively cheap. Even so, Bates [11] relates that in 1901 the industry reduced the number of section sizes into a standardized list (a forerunner of the modern catalogue: SCI [22]) to reduce costs. During the Second World War, Beal [11] reports that safety factors were reduced to ‘economise on scarce materials’, suggesting that material costs still outweighed those for labour.

Gibbons [23] states that since the 1960s ‘the cost of plain steel sections in the UK has decreased dramatically relative to the unit cost of labour’, with the ratio of fabrication labour costs to material costs rising from 1 in 1960 to 2.88 by 1990. This change caused Needham [24] to advise that ‘only rarely does [minimum weight design] achieve lowest cost’. Both He & Gibbons [23] describe how a design using a small number of different section sizes in a repetitive configuration with few alterations required to the sections is easier to detail, fabricate and construct, thus saving labour, hence cost, despite weighing more. Gibbons terms this practice ‘rationalization’ and SCI [25] gives further guidelines for it, noting that procurement costs are also reduced by large, repetitive orders resulting from rationalization.

Needham and Gibbons estimate the threshold of weight increase beyond which rationalization gives no cost saving at 5–10% and 20%, respectively, though neither provides justification for their value, and no published study has been completed examining the validity of these estimates. In the only published study of the efficiency of built designs, Sadek *et al.* [26] analyse six concrete residential structures in Kuwait—a country with different material and labour costs to the UK—finding some contain twice as much reinforcement as necessary which increases costs; they attribute this to poor design practice rather than rationalization, however.

### (c) Conclusions from literature review

Published literature on the provision of structural steel in buildings reveals that it has become possible to use less material to support the same loads safely due to increasing human knowledge of steel production and performance; there remains further scope to use less material still. However, strategies to produce economic structures have changed in the UK as the ratio of material to labour cost has changed in construction, with current practice favouring rationalization as a method of achieving lower costs by adding extra material (where this allows a reduction in labour costs). This extra material does not supplement structural performance, it does not enhance safety and it is merely surplus material. To date, there is no published analysis quantifying the extra mass actually added due to rationalization. This article therefore puts forward a method to do so, and applies it to an extensive dataset gathered from recent practice, permitting estimation of the potential for steel saving from decreasing rationalization, and the associated reduction in carbon dioxide emissions.

## 3. Methodology to analyse utilization of structural steel elements

A methodology is proposed to analyse steel elements in building designs to infer the potential to reduce steel mass without adversely affecting performance. It is based on the concept of a ‘utilization ratio’ for structural elements, already used in the industry, as described in §3*a*, and applied to the beams (§3*b*) and columns (§3*c*) of 23 commercially designed, steel-framed buildings supplied by three leading UK design consultancies. All factors influencing these designs were not known; therefore, a verification process, described in §3*d*, was used to ensure the results were reflective of the reality of each building's design.

### (a) Utilization of structural steel

A ‘utilization ratio’ (abbreviated to U/R; also called ‘utiliation factor’ or ‘unity factor’) is defined in equation (3.1) as the ratio of the actual to maximum allowable performance values.

3.1 $$\text{utilization ratio}=\frac{\text{actual performance value}}{\text{maximum allowable performance value}}.$$

This ratio can be calculated for a range of performance requirements for steel elements (beams or columns). For any element, engineers are concerned with the highest U/R across all performance requirements, as this defines the most probable failure; for example, for bending moments, the numerator of equation (3.1) is the largest applied moment along the element and the denominator is the element's moment capacity. Design standards such as Eurocode 3 contain equations of this type for all performance requirements of interest, with specific calculation instructions and specify the maximum value of the ratio (usually 1) [13].

By determining the maximum utilization of an element, a U/R also indicates its excess capacity—i.e. the material that is unnecessary. By analysing U/Rs for an entire building the total potential steel saving can be estimated. For simplicity of calculation, it was assumed that steel requirements were directly proportional to U/R for both composite and non-composite elements. To ascertain an average level of saving potential, a substantial dataset was assembled by collecting structural design data for steel-framed, commercially designed buildings, supplied by three leading UK design consultancies. The selected buildings were ‘typical’ UK steel-framed buildings, excluding those with unusual geometries, very long spans or particularly onerous design conditions, and were mainly schools, offices and residential buildings. In total, information on 12 787 beams and 2347 columns of 23 building designs was obtained and analysed using 12 man-months of labour over a 2-year period. As each building design is bespoke, this large dataset was necessary to facilitate statistical analysis to determine average values and prevent results being skewed by individual buildings.

Six different design criteria were examined for each element: moment, shear, axial force, buckling, combined axial and moment buckling, deflection (full details are included in the electronic supplementary material). These criteria were chosen as design guidance mandates that they be checked and they can be meaningfully expressed as a U/R with maximum value 1.0. For each element, the U/R for each criterion was calculated at the most onerous point according to the design standard originally used for the parent building (Eurocode 3 [13] or British Standard 5950 [27]). The highest U/R was then selected as the single, governing U/R for the entire element, and this was used in subsequent analysis.

Data were assumed valid and accurate unless they indicated overload or bracing, or a U/R could not be calculated. Elements were omitted from the analysis if they had U/Rs in excess of 1.00 (i.e. over-loaded and thus failing) because it was assumed that such elements were later corrected to non-failing sections, but without knowledge of the new sections it was impossible to calculate a U/R. Elements designated as bracing (or those with U/Rs equal to zero, assumed to be bracing) were omitted because U/Rs are not meaningful for such elements. Elements for which there was not enough information to calculate a U/R were also omitted from the analysis. In total, 2657 beam and 510 column data were omitted; insufficient information was the main reason for omitting columns while the majority of beam omissions were caused by overload. The valid dataset therefore consisted of 10 130 beams and 1837 columns. Table 1 summarizes the dataset assembled.

**Table 1.** Summary of building steel dataset.

			beams		columns	
building number	building type	floors analysed	total	omitted	total	omitted
1	office	3	186	39	52	2
2	hospital	2	802	23	156	9
3	school	3	106	3	30	0
4	school	2	62	0	21	0
5	office	1	21	0	15	0
6	office and education	3	1194	494	75	0
7	school	5	908	142	113	10
8	office	5	519	144	40	2
9	office	4	606	94	59	3
10	office	1	48	13	0	0
11	school	3	503	124	109	55
12	school	2	578	52	108	8
13	school	1	372	61	74	10
14	school	3	760	9	168	2
15	residential and retail	14	2230	783	215	147
16	mixed-use residential	6	536	172	215	154
17	mixed-use residential	8	947	316	164	99
18	office	2	316	116	57	0
19	school	3	527	28	151	1
20	school	2	322	8	96	1
21	residential	1	73	2	213	0
22	school	3	613	8	118	7
23	school	2	558	26	98	0
	totals	79	12 787	2657	2347	510

### (b) Utilization of beams

A statistical analysis of the beam data was undertaken to find patterns in, and hence draw conclusions about, the utilization of steel in each building. The entire 23-building dataset was also analysed to determine what ‘typical’ utilization was. The valid U/R data for the beams in each building were averaged to produce an estimate of how much spare capacity the building had. U/Rs were then averaged by beam length and mass to determine variance with these characteristics. The frequency of occurrence of each U/R within the building was calculated and graphed, grouping U/Rs in 10% bands for clarity. It was assumed that beams which were explicitly designed would have U/R greater than 0.8, so this proportion was calculated for each building. Beams with U/R below 0.2 are unlikely to have been designed, so this proportion was also found. As buildings are designed floor-by-floor, U/Rs were also averaged and graphed by frequency for floors with large numbers of beams—beams from small floors or otherwise miscellaneous were classified as ‘other’ when reported and are included in the building ‘total’ graph line, always marked in black. It was noticed that many floorplates used a small number of the sections available in SCI's standard catalogue [22] so the five most common section sizes were identified for each floor and their proportion of the total number of beams on that floor calculated.

**Figure 1.**
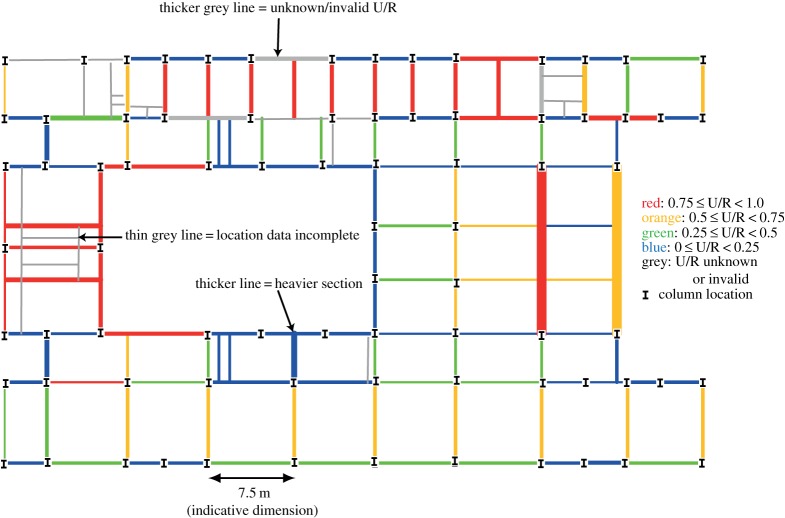
Example plot of floor showing U/R and section weight. (Online version in colour.)

To understand how U/Rs varied across the layout of each floor of each building, plots were produced showing the U/R of each beam, an example of which is shown in figure 1. This plot is a plan-view of a floor, just showing the beams and column locations. The beams are coloured according to their U/R as indicated in the legend of the figure. The thickness of each line is proportional to the beam's linear weight (kg/m). Where a beam's U/R was not available or omitted, its line was coloured grey. For many of buildings, the beam location data were incomplete; in these circumstances, beams were manually added in thin grey lines to convey the floor geometry and beam layout. An indicative dimension shows the scale of each floor. Sufficient location data were available to produce 43 such plots for floors of 17 buildings, all of which are contained in the electronic supplementary material.

### (c) Utilization of columns

U/R data for the columns in 22 buildings were also analysed to identify patterns and determine typical values. Average U/Rs were calculated and graphs were plotted showing the frequency of U/Rs. However, no data were available on column mass, length or location which prevented further analysis of these elements.

### (d) Verification processes

The six design criteria analysed do not form an exhaustive list; there are other criteria which influence the size of element chosen. To determine the reasoning behind the designs and hence understand these omitted criteria, a semi-structured interview was held with a structural engineer for each building. Each engineer was asked the following questions, derived from discussions with experienced industry professionals:

— The information supplied was for which building design stage? Did the constructed steelwork vary significantly from this information?
— Apart from design criteria analysed, did any other criteria govern? For example: vibration requirements, construction loads/considerations, section depth limits; if so, where?
— How was robustness provided for in the design? Did it change element sizes? Are there any other factors which are of interest to this study?

A copy of the interview form used, containing further detail, is supplied in the electronic supplementary material. Interview responses were analysed with the analytical data to determine correlations and explore causations.

The catalogue of standard hot-rolled structural steel sections [22] was analysed to determine the reduction in capacity between consecutive sections—a practical limitation on the U/R achievable in design. Moment, shear and axial capacity were calculated using Eurocode 3-1-1 [13] for each Universal Beam and Universal Column section; bending stiffnesses were also calculated. These four properties were compared with those for the beam one size larger and the reductions in each calculated; the average reduction was calculated using the largest of these reductions.

## 4. Results from analysis of steel utilization

The methodology described in §3 was applied to the data from the 23 steel-framed buildings. Full results are detailed in the 66 pages of electronic supplementary material containing 88 figures and 23 tables. A summary presented in §4*a*, reveals that steelwork is not used as expected. The reasons for this are explored in §4*b*, where a practically achievable average U/R for buildings is proposed.

### (a) Utilization results

A summary of the results of the analysis of U/Rs of beams in 23 buildings is presented in table 2, including results averaged across all buildings. The average U/R across all projects is 0.40, with a range of 0.15–0.90. The average rises when weighted by length—to 0.48—and again by mass, to 0.54, with a range of 0.25–0.96. On average, twice as many beams have U/Rs less than 0.20 as do greater than 0.80, while the five most common beam sections in a building account for three in every four beams typically. Results for the average U/R of columns in each building are also listed in table 2, showing an overall average of 0.49 with a range of 0.12–0.72.

**Table 2.** Results of the analysis of utilization ratios for beams and columns in buildings.

		avg. U/R for beams			% beams with U/R			
building number	no. valid beams	by piece	by length	by mass	≤0.20	>0.80	five most common beams %	columns avg. U/R
1	147	0.36	0.47	0.43	39	7	66	0.31
2	779	0.58	0.62	0.68	16	30	68	0.60
3	103	0.25	0.32	0.47	65	4	90	0.12
4	62	0.17	0.37	0.62	75	5	66	0.13
5	21	0.44	0.41	0.41	0	0	100	0.64
6	700	0.15	0.21	0.25	69	0	77	0.42
7	766	0.33	0.39	0.45	42	9	62	0.47
8	375	0.31	0.39	0.39	43	7	81	0.72
9	512	0.37	0.49	0.50	38	16	49	0.60
10	35	0.90	0.93	0.96	0	83	100	—
11	379	0.64	0.70	0.68	12	45	71	0.69
12	526	0.47	0.56	0.60	32	17	64	0.49
13	311	0.39	0.48	0.49	38	17	75	0.52
14	751	0.26	0.32	0.38	56	3	65	0.54
15	1447	0.18	0.27	0.37	75	4	74	0.62
16	364	0.23	0.37	0.46	51	9	95	0.57
17	631	0.52	0.65	0.70	26	35	81	0.60
18	200	0.54	0.68	0.66	27	40	97	0.12
19	499	0.36	0.39	0.43	50	15	71	0.49
20	314	0.33	0.47	0.66	58	25	74	0.35
21	71	0.55	0.54	0.61	0	17	99	0.65
22	605	0.47	0.58	0.63	28	20	78	0.55
23	532	0.35	0.44	0.50	50	11	87	0.60
average	440	0.40	0.48	0.54	39	18	78	0.49

**Figure 2.**
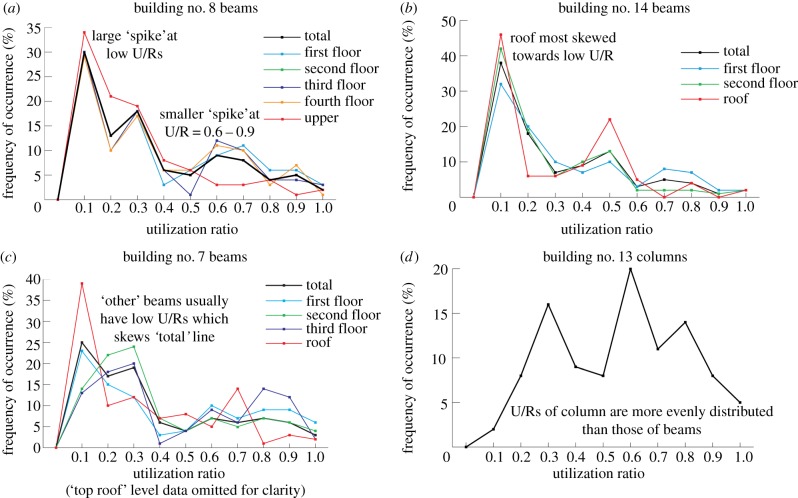
Four of 45 graphs of frequency occurrence against utilization ratio, three for beams (by floor and overall) and one for columns, displaying patterns found across all buildings. (Online version in colour.)

Frequency graphs were plotted for all buildings and are contained in the electronic supplementary material. Figure 2 displays four graphs that exhibit the following patterns found across the entire dataset: a large ‘spike’ at low U/R with a smaller spike between U/Rs of 0.6 and 0.9 (figure 2*a*); this spike pattern does not vary significantly depending on floor, although roofs more frequently have larger low U/R spike (hence lower average U/R) despite often containing the largest number of beams (figure 2*b*); the building ‘total’ line is often skewed towards low U/Rs because this line includes the ‘other’ beams from small floors and miscellaneous areas which have consistently lower U/Rs (figure 2*c*). Graphs of frequency against U/R for columns, as provided in the electronic supplementary material, have a flatter frequency distribution (i.e. less prominent spikes) than for beams typically (figure 2*d*). Perhaps surprisingly the results show no correlation between building type and utilization.

**Figure 3.**
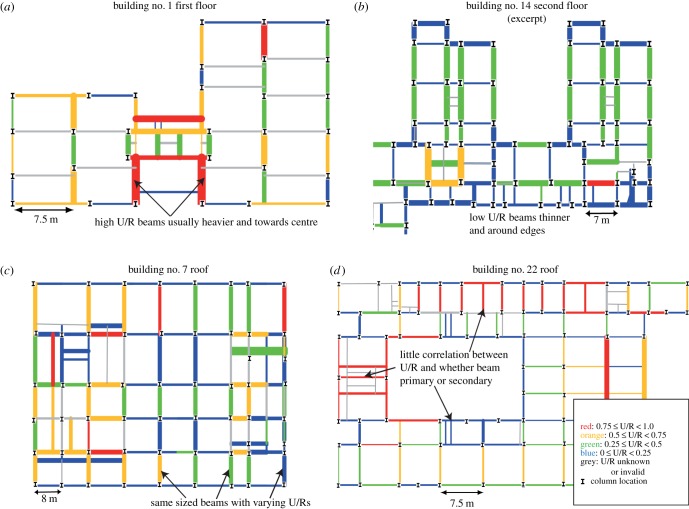
Four of 43 plots that indicate beams’ U/R and section weight. These examples show typical patterns found across entire dataset. (Online version in colour.)

Figure 3 shows four floor plots of U/R and beam mass which exemplify the patterns found across the 43 plots produced: beams with high U/R (coloured in red) are generally located towards the middle of buildings and tend to have larger section sizes (i.e. thicker line weights), while beams with low U/R (coloured in blue) are lighter (thinner lines) and situated more often around the periphery (figure 3*a*,*b*, respectively); beams of the same section size (i.e. line thickness) near to one another often display a range of colours (figure 3*c*); whether a beam was loaded by other beams (i.e. whether it was a primary beam or not) did not appear to correlate strongly with its U/R (figure 3*d*).

### (b) Verification results

Table 3 summarizes the responses from the interviews with designers; further details are noted alongside the relevant building results in the electronic supplementary material. All building information was at or close to the construction stage, and nowhere did robustness significantly impact element sizes. Each building was designed to a specific brief for a unique site at a precise time, but nonetheless recurring themes were present in the responses. As can be seen in the table, engineers reported that floor vibration requirements (a criterion not included in the analysis) governed beam design in large areas of three buildings and small areas of a further six designs, thus affected elements’ true U/Rs will be higher. However, the former three buildings have average U/Rs by mass *higher* that the average across all buildings, suggesting that vibration does not directly lead to low U/Rs.

**Table 3.** Summary of responses from interviews with designers.

	criteria that governed steel design:			
building number	vibration?	construction?	section depth?	further detail and comments
1	no	yes	no	torsion during construction; larger beam sizes the cheapest solution
2	no	no	no	no special criteria
3	no	no	no	deflection governed mainly
4	no	no	no	deflection governed mainly
5	no	no	no	applied loads reduced but too late to redesign
6	small areas	yes	small areas	perimeter beams governed by vibration or connection depth
7	small areas	no	no	mainly stress and deflection governed
8	no	no	no	many omitted beams were fabricated bespokely
9	small areas	no	no	many omitted beams were fabricated bespokely
10	no	no	no	had time to design thoroughly and no late changes
11	no	no	no	steelwork rationalized to reduce procurement costs
12	no	no	yes	building geometry optimized to reduce facade and heating costs
13	no	no	no	steelwork rationalized to reduce procurement costs
14	small areas	yes	small area	torsion during construction; larger beam sizes the cheapest solution
15	no	no	no	complex procurement procedure increased steel tonnage
16	no	no	no	complex procurement procedure increased steel tonnage
17	large areas	no	yes	shallow beams used to minimize cladding costs
18	large areas	no	yes	shallow beams used to minimize cladding costs
19	no	no	no	deflection governed most beam designs
20	large areas	no	no	vibration governed in many areas
21	small areas	no	yes	standardized beam depths for architectural reasons
22	no	yes	no	steelwork rationalized to allow faster construction
23	one area	yes	no	sizes repeated to allow faster construction

Cost concerns featured prominently in the interview responses: designers deliberately repeated section sizes for economies of scale during procurement and to reduce mix-ups on site, and used larger sizes than necessary to facilitate easier connection and hence reduce labour requirements during construction. Despite this, most designers were surprised that average U/Rs were so low: a number of them suggested that maximum depth limits on beams lead to low U/Rs, however, one engineer noted that a shallow section should still have a high U/R despite being heavier than a deeper section.

The results from analysis of the catalogue of steel sections revealed that on average a Universal Beam section has 85% of the capacity of the beam one size larger, whereas for a Universal Column section the corresponding value is 81%. Assuming that U/Rs are uniformly distributed between these values and 1.00, an average U/R of 0.9 is therefore possible using this catalogue.

## 5. Discussion of implications from results

The surprising results of §4 reveal that buildings could potentially be designed with around half the steel used at present and still deliver specified safety and service levels to occupants. The results point towards rationalization as the main cause of this over-specification of steel beams in construction, and this has implications for three groups within the construction industry: designers; contractors and fabricators; clients, standards committees and policy-makers.

### (a) Evidence for rationalization as the cause of low utilization

The result that the average utilization of beams weighted by mass is 0.54 implies that up to 46% of the buildings’ combined beam mass is not necessary (relative to the design standards and before any novel optimization). An average U/R of 0.9 is possible with the discrete catalogue of section sizes, thus 36% of a building's beam mass could be removed with no loss in safety or service. Comparing the results from §4 with the descriptions of rationalization in §2.2, the following observations are made:

— low variation of section sizes in a floor or building: the five most common sections make up almost three-quarters of the beams in a building, and usually over 80% of the beams on a floor, regardless of area; this suggests repetition in design;
— low frequency of high U/Rs: fewer than one beam in five has a U/R greater than 0.8 (the assumed lower bound for elements explicitly designed by an engineer) which suggests that most beams are not explicitly designed;
— sections being copied across a floor: the floor plots showed high and low U/Rs for beams with the same section size near each other, supporting the previous point that beams are copied across from the most onerous scenario (explicitly designed) to less onerous ones (which then have low U/R);
— recognition by designers that cost concerns cause repetition of section sizes and use of larger section sizes than necessary.

These four points indicate that rationalization is occurring and is the most likely cause of U/Rs being lower than expected. Interestingly, there appears to be no correlation between the number of beams in a building and its average U/R—rationalization affects big buildings as much as small ones.

The average U/R by piece for columns is 0.49 compared with 0.40 for beams—it is not clear why columns are more highly used by piece than beams. It may be they are simpler to design, being mainly axially loaded or that there are fewer of them, or that this analysis included all design criteria for columns and that beams’ average U/R would appear to be higher if omitted criteria such as vibration were included. As column pieces are usually two to three storeys in length, the bottom storey is most heavily loaded and will govern design, therefore, the upper parts of each piece could have very low utilizations.

### (b) Implication of results for designers

The interviews confirmed that engineers are responding to clients’ requests for low-cost buildings by following the rationalization guidelines outlined in §2.2. However, designers are not aware that rationalization is adding almost 40% extra steel mass to the building, four times what Needham [24] suggests and double what Gibbons [23] recommends. While it is not clear whether this level of rationalization is economically beneficial, it is certainly contributing unnecessarily to carbon dioxide emissions through production of unnecessary extra steel.

Rationalization can be reduced by at least two methods: by increased the time engineers have to design buildings, or by greater use of existing steelwork design and optimization software. Both strategies involve extra cost but reductions in steel mass may offset these, particularly as weight savings compound—i.e. a lighter floor-structure requires less column material to support it and thus smaller foundations—particularly for tall buildings. Benefit can be maximized from both strategies by: targeting areas of low U/R such as roofs, half-levels and ‘miscellaneous’ elements; focusing on beams before columns (Moynihan & Allwood [9] note that beams contain three times as much steel mass as columns when aggregated across a building); checking light or short beams (that average U/R by length and mass are higher than by piece indicates these beams have low U/Rs). Computer software offers further potential to decrease steel through use of more complex design methods. Simply calculating the average U/R for a building may spur designers to increase it in an economic way—Needham [24] notes the satisfaction engineers derive from finding an optimum design. It is noteworthy that building #10 achieved an average U/R in excess of 0.9 within existing commercial constraints.

### (c) Implications for contractors and fabricators

Literature on economic design, reviewed in §2*b*, reveals that many rationalization measures are motivated by fabrication and construction considerations, thus increased use of technology in the fabrication factory and on site could reduce the incentive to rationalize. Increased automation and flexibility of fabrication could reduce the amount, hence cost, of labour to fabricate sections and allow many different section sizes to be processed with little additional cost. Increased use by contractors of information technology on site (such as radio tags described by Ikonen *et al.* [28]) could reduce the motivation to standardize beam sizes if there is less risk of pieces being placed or installed incorrectly. Cost structures within the industry may need adjustment to ensure profit and material reduction motives are aligned—for instance, fabricators have no incentive to reduce steel mass when paid per tonne, but might if rewarded with a proportion of the material cost saved. Rationalization by fabricators and contractors (after that by the designer) is not included in this study; a number of interviewees remarked this rationalization is anecdotally larger than that done by designers, but without ‘as built’ information for each building it was not possible to verify this.

### (d) Implications for clients, standards committees and policy-makers

As noted in §5*b*, designers respond to the instructions of their clients so environmentally minded clients, or those who simply do not like waste, could reduce excess material in the buildings they commission by specifying a minimum average U/R. A value in the region 0.70–0.90 will give environmental benefit while being practically achievable. Clients can be motivated by sustainable building schemes, such as BREEAM in the UK or LEED in the USA, so these should consider adding credits for achievement of minimum average U/Rs as incentives to reduce rationalization. Designers usually work within design standards, so those who set them should consider not just having a maximum U/R for elements (as currently is the case) but also a minimum U/R, or a target U/R range below which justification is required; local authorities might also make planning permission contingent on similar metrics being achieved. An initial step for the latter three parties may be to mandate reporting of average U/R.

### (e) Potential benefits and future research

The 10 130 beams analysed cumulatively contain 2823 tonnes of steel. Presuming the average U/R by mass for this steel could be raised from 0.54 to 0.90, this would have avoided use of 1027 tonnes of steel. Applied to the 290 million tonnes of steel used worldwide to construct buildings each year [9] this 36% reduction would save 106 million tonnes of steel per year and would avert 214 million tonnes of carbon dioxide emissions (based on 2.03 kgCO_2_ kg^−1^ from Hammond & Jones [29]).

Further research would be required to obtain a more precise estimate of steel saving, accounting for the nonlinear relationship between U/R and material provision—for instance, a deeper section may have twice the moment capacity without having twice as much mass; this phenomenon is amplified for composite construction. Research is also required to understand the economics of rationalization, as current levels of excess steel are double what was estimated as the upper threshold for net saving. Specifically, it is not known how much more design time is required to achieve a percentage increase in average U/R, nor what the extra cost of fabricating and constructing this design would be, nor how these extra costs compare with the saving in material cost and with the overall project cost. Studies of utilization could be undertaken for different buildings types (e.g. portal frames, which account for one-third of construction steel use [9]) or different structural materials (e.g. reinforced concrete or timber) to assess further opportunities to use less material in construction.

## Supplementary Material

Utilisation of structural steel in buildings SI
