# Supplementary material for: Utilization of structural steel in buildings
Source: Proc Math Phys Eng Sci. 2014 Aug 8;470(2168):20140170. doi: 10.1098/rspa.2014.0170 (PMC4075790; doi:10.1098/rspa.2014.0170)
Supplement: Utilisation of structural steel in buildings SI [file rspa20140170supp1.pdf]

Utilisation of structural steel in buildings: supporting information

Muiris C. Moynihan and Julian M. Allwood*

Cambridge University Engineering Department, Trumpington Street, Cambridge, CB2 1PZ, UK.
*Corresponding author email: jma42@cam.ac.uk; telephone: +44-1223 338181.

This document contains supplementary information as described in the journal article "Utilisation of structural steel in construction". It is divided into 3 sections:

- Section 1 contains results for each of the 23 buildings analysed as per section 4 of the journal article;
- Section 2 contains details on the design criteria included when calculating utilisation ratios, referenced in section 3.1 of the journal article;
- Section 3 contains the list of questions used when interviewing building designers, referenced in section 3.2 of the journal article.

SECTION 1: BUILDING DATA

This section details the results for each of the 23 buildings analysed. As agreed with the providers of the raw data, each building is identified only by a number, with the following information provided:

- Building type;
- Number of beam data obtained and number analysed;
- Table with summary of results by floor and overall;
- Graph of frequency of occurrence against utilisation ratio for each floor and overall;
- Plot of beam layout on each floor analysed showing utilisation ratio of each beam;
- Graph of frequency of occurrence against utilisation ratio for the columns in the building.

For all buildings it was possible to provide the first four items. However limitations in the data resulted in three categories of building for the remaining two items:

- For 17 buildings over 70% of the beams on each floor could be plotted, and once this level was reached the floor was deemed finished, as patterns were clear. Where necessary to complete the floor geometry, and so aid comprehension of the data, omitted beams were added in manually (coloured grey). Column locations were also added manually for this reason.
- For 6 buildings (#s 8, 9, 11, 16, 17, 21) there was insufficient information on beam layout to produce plots;
- For 1 building (# 10) there was insufficient information to produce a graph of column data.

For graphs, utilisation ratios are groups into bands of 10% to aid clarity; these bands are inclusive of the identifying upper bound, for example the data point at 0.2 includes U/Rs from 0.11 to 0.20.

For all plots of beam utilisation ratio per floor the legend below is used:

Legend
$0.75 \leq U/R < 1$
$0.5 \leq U/R < 0.75$
$0.25 \leq U/R < 0.5$
$0 \leq U/R < 0.25$
U/R invalid or unknown
I Column

Building #1

Type: office

147 of 186 beams analysed (79%)

Table 1: summary of results by floor for building #1

Level	No. beams analysed	% of total steel mass	Avg. U/R	Weighted avg. U/R	Top 5 Beams No.	Top 5 Beams %
Roof	15	3%	0.23	0.34	-	-
Plant room	54	55%	0.49	0.65	35	65%
1st floor	52	42%	0.39	0.57	30	58%
**TOTAL**	**147**	**100%**	**0.36**	**0.43**	**98**	**66%**

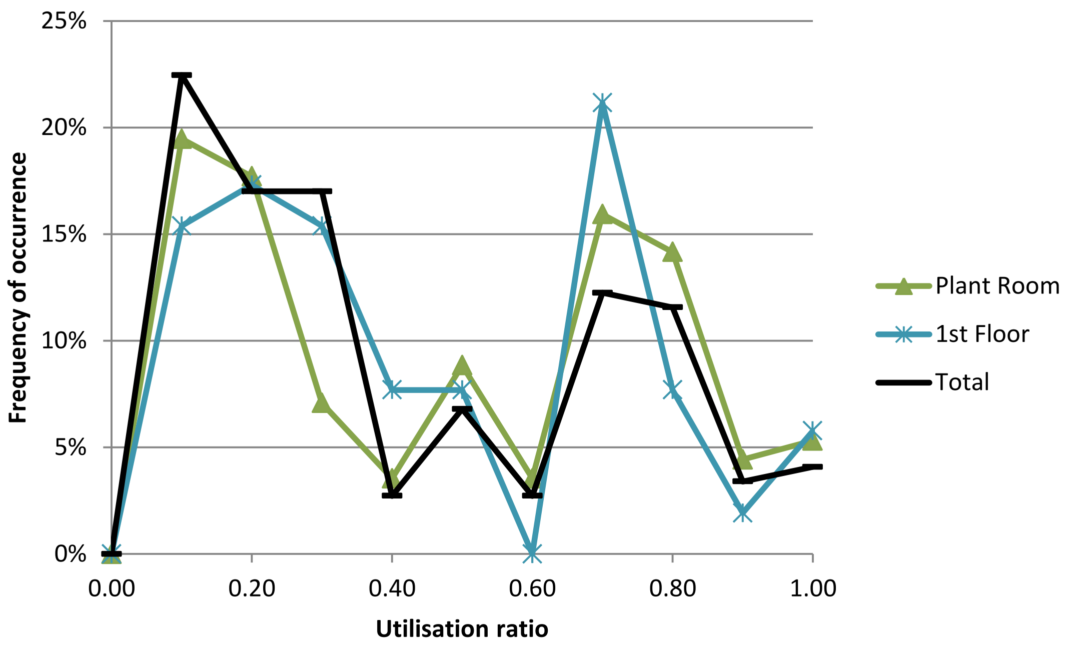

Figure 1: graph of frequency of occurrence against utilisation ratio for beams by floor and overall for building #1

1st floor

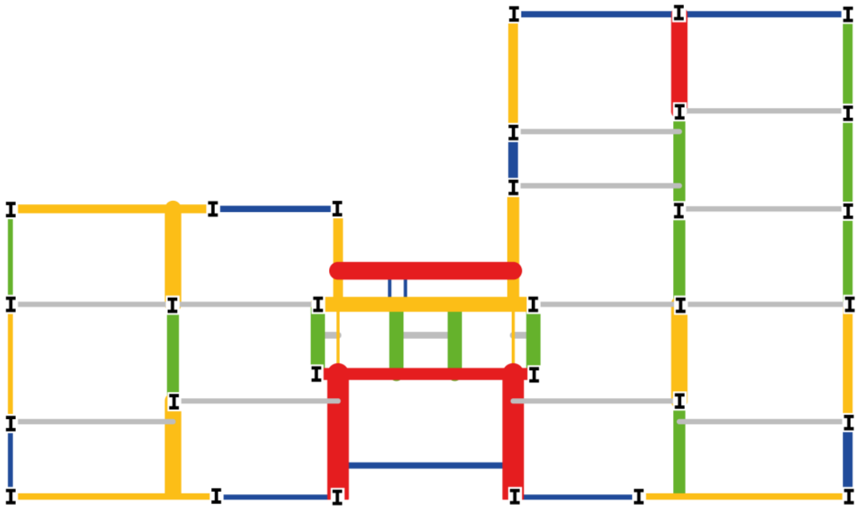

Figure 2: plot of floor showing beams coloured according to utilisation ratio (as per legend pg. S2)

Plant level

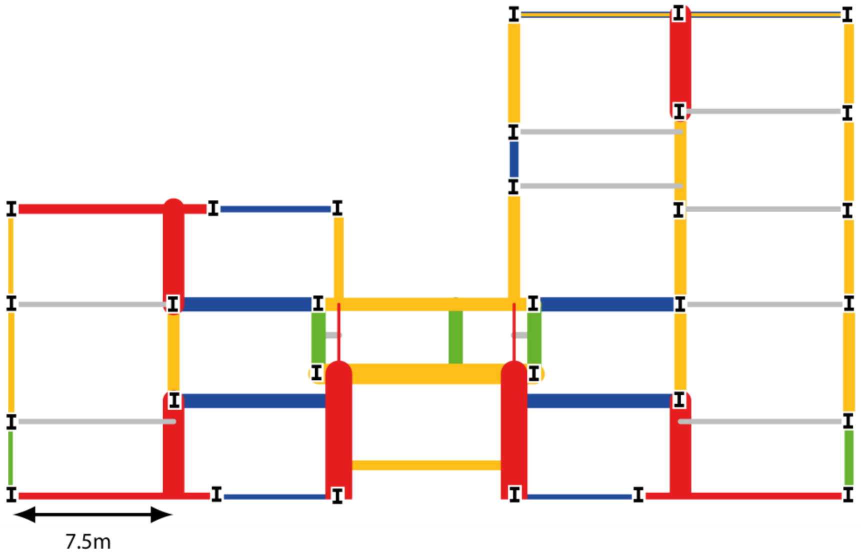

Figure 3: plot of floor showing beams coloured according to utilisation ratio (as per legend pg. S2)

Engineer's comments

A proportion of beams governed by construction loading scenario, otherwise a standard building. A spot-check of beam sizes did not reveal any further rationalisation by fabricator. Robustness was not a governing criterion.

Columns

50 of 52 columns analysed (96%)

Average U/R: 0.31

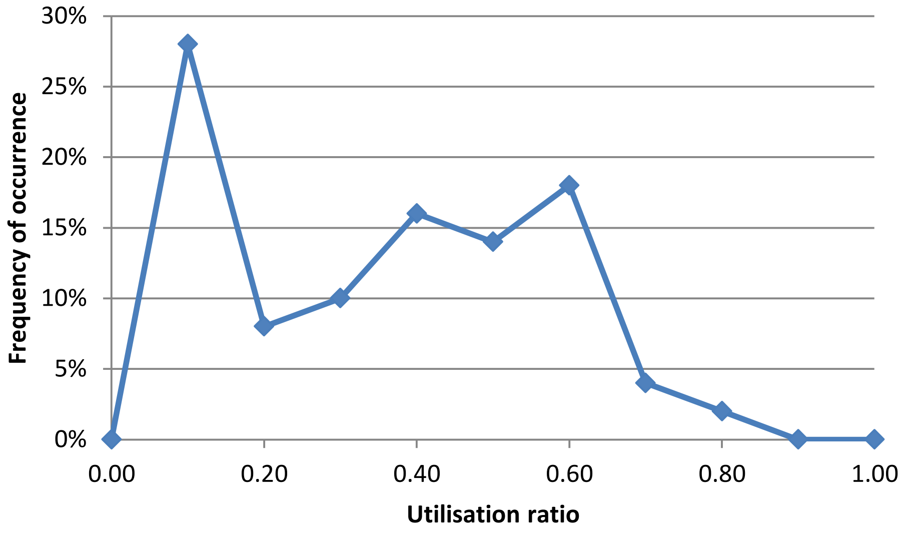

Figure 4: graph of frequency of occurrence against utilisation ratio for columns by floor and overall for building #1

Building #2

Type: hospital

779 of 802 beams analysed (97%)

Table 2: summary of results by floor for building #2

Level	No. beams analysed	% of total steel mass	Avg. U/R	Weighted avg. U/R	Top 5 Beams No.	Top 5 Beams %
2nd floor	236	30%	0.57	0.69	215	91%
1st floor	327	50%	0.70	0.73	272	83%
Other	*216*	*20%*	*0.42*	*0.52*	-	-
**TOTAL**	**779**	**100%**	**0.58**	**0.68**	**532**	**68%**

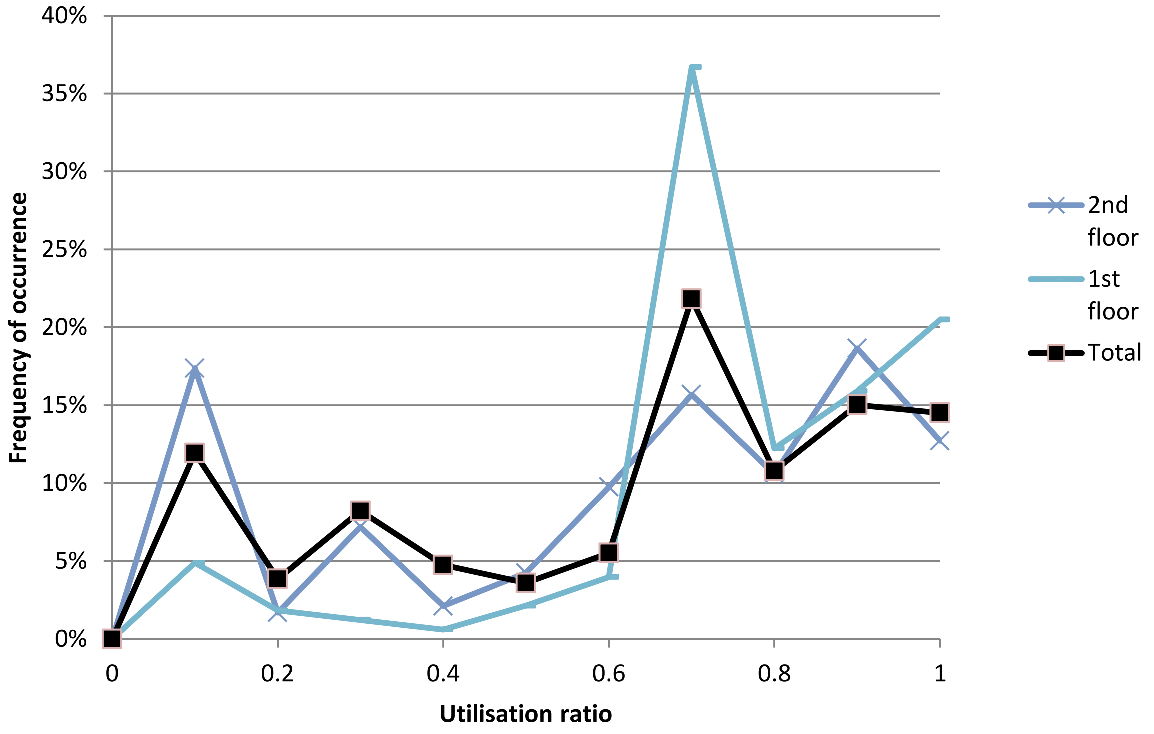

Figure 5: graph of frequency of occurrence against utilisation ratio for beams by floor and overall for building #2

Only one foundation drawing was available to base the below plots on – therefore column locations have been inferred and 'missing' beams added according to engineering intuition.

1st floor

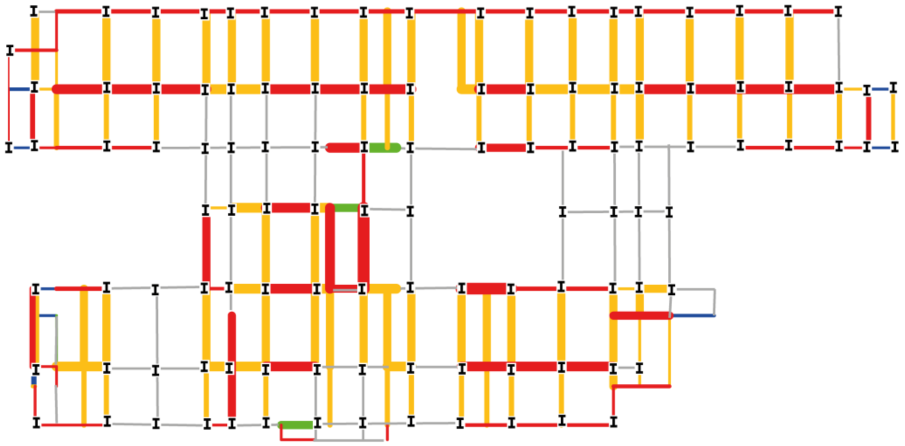

Figure 6: plot of floor showing beams coloured according to utilisation ratio (as per legend pg. S2)

2nd floor

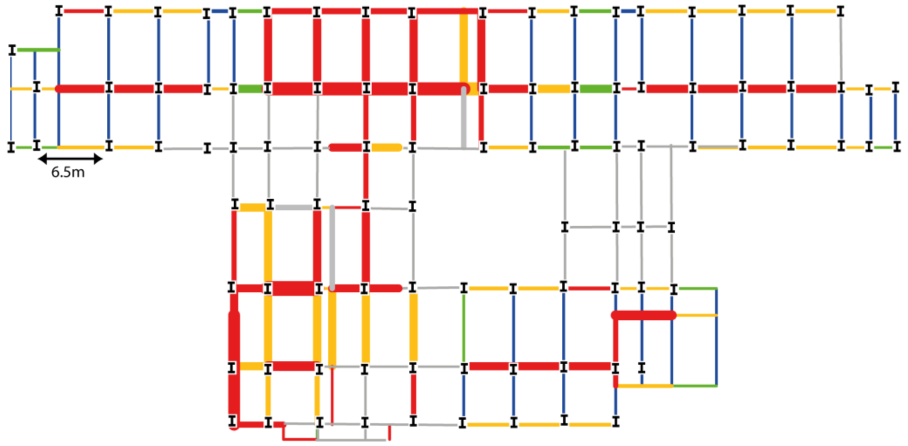

Figure 7: plot of floor showing beams coloured according to utilisation ratio (as per legend pg. S2)

Engineer’s comments:

No special vibration or other requirements.

Columns

147 of 156 columns analysed (94%)

Average U/R: 0.60

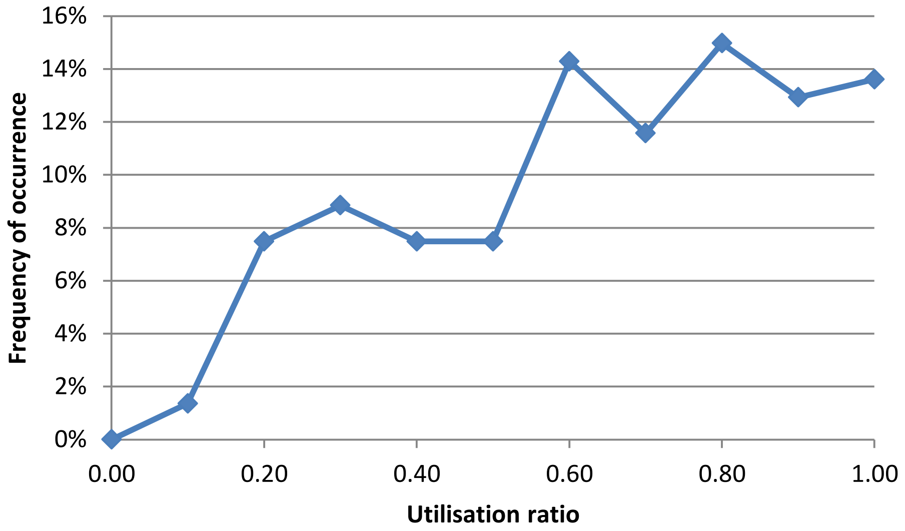

Figure 8: graph of frequency of occurrence against utilisation ratio for columns by floor and overall for building #2

Building #3

Type: school

103 of 106 beams analysed (97%)

Table 3: summary of results by floor for building #3

Level	No. beams analysed	% of total steel mass	Avg. U/R	Weighted avg. U/R	Top 5 Beams No.	%
Roof	8	32%	0.35	0.63	8	100%
Plant Roof	26	24%	0.22	0.44	26	100%
1st Floor	26	20%	0.22	0.53	26	100%
Other	*43*	*24%*	*0.27*	*0.19*	-	-
**TOTAL**	**103**	**100%**	**0.25**	**0.47**	93	90%

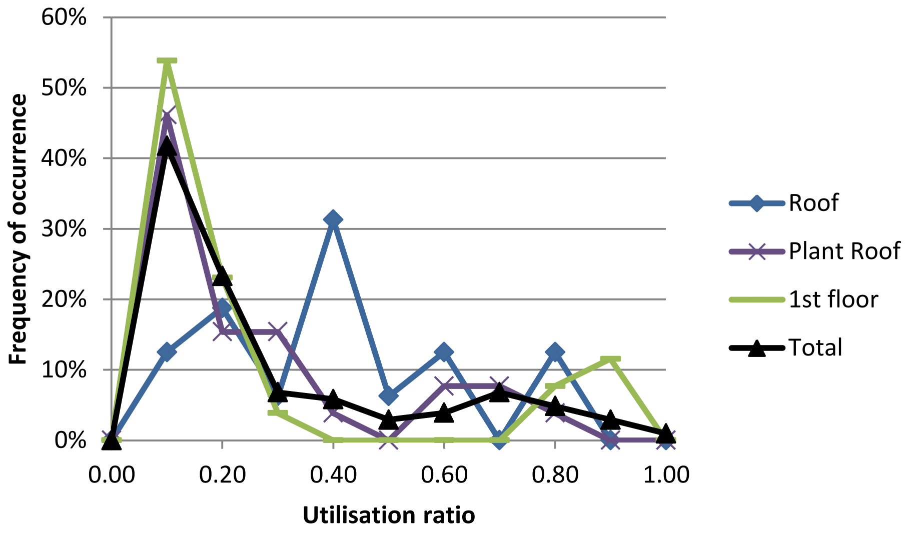

Figure 9: graph of frequency of occurrence against utilisation ratio for beams by floor and overall for building #3

1st floor

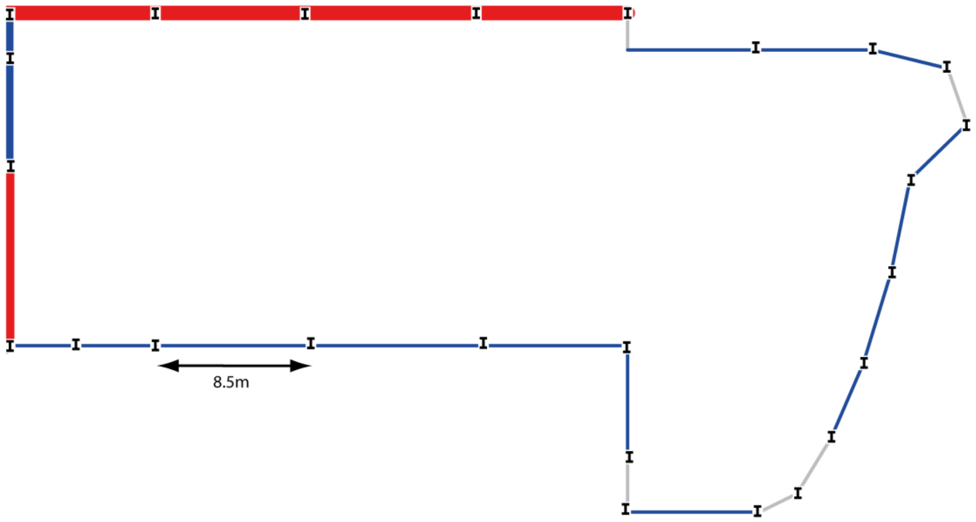

Figure 10: plot of floor showing beams coloured according to utilisation ratio (as per legend pg. S2)

Plant roof

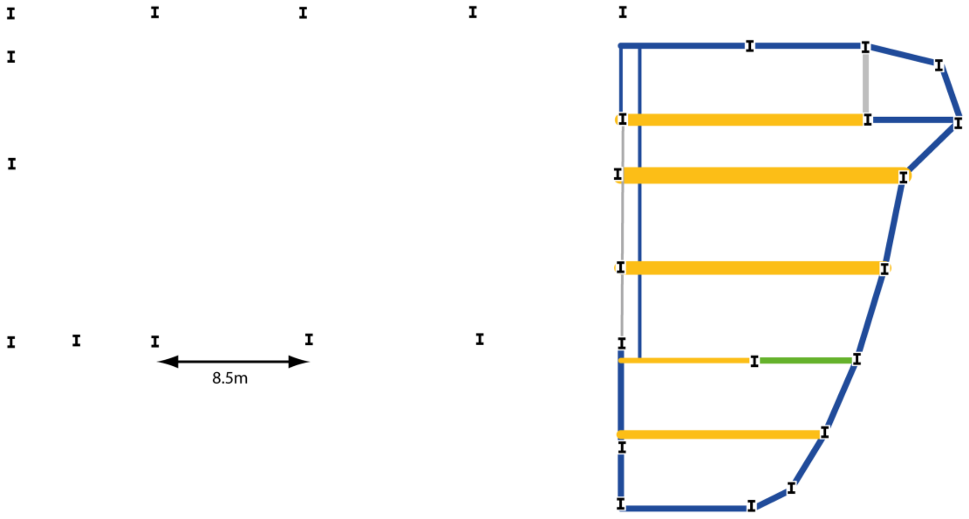

Figure 11: plot of floor showing beams coloured according to utilisation ratio (as per legend pg. S2)

Roof

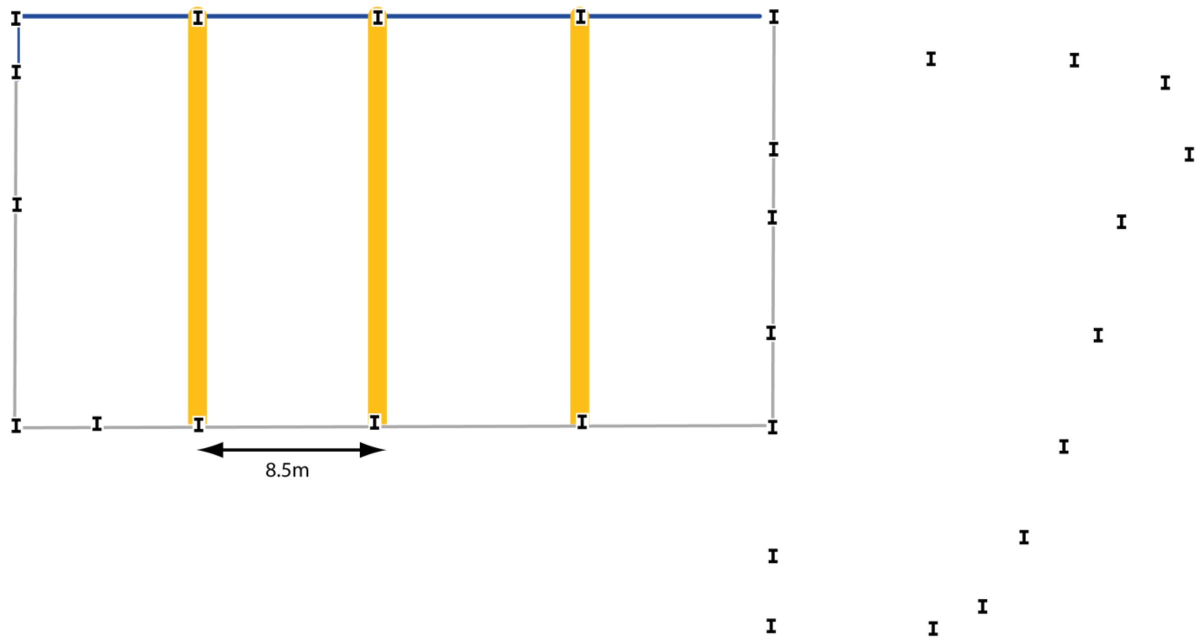

Figure 12: plot of floor showing beams coloured according to utilisation ratio (as per legend pg. S2)

Engineer's comments

Design deflection governed primarily; robustness not an issue.

Columns

30 of 30 columns analysed (100%)

Average U/R: 0.12

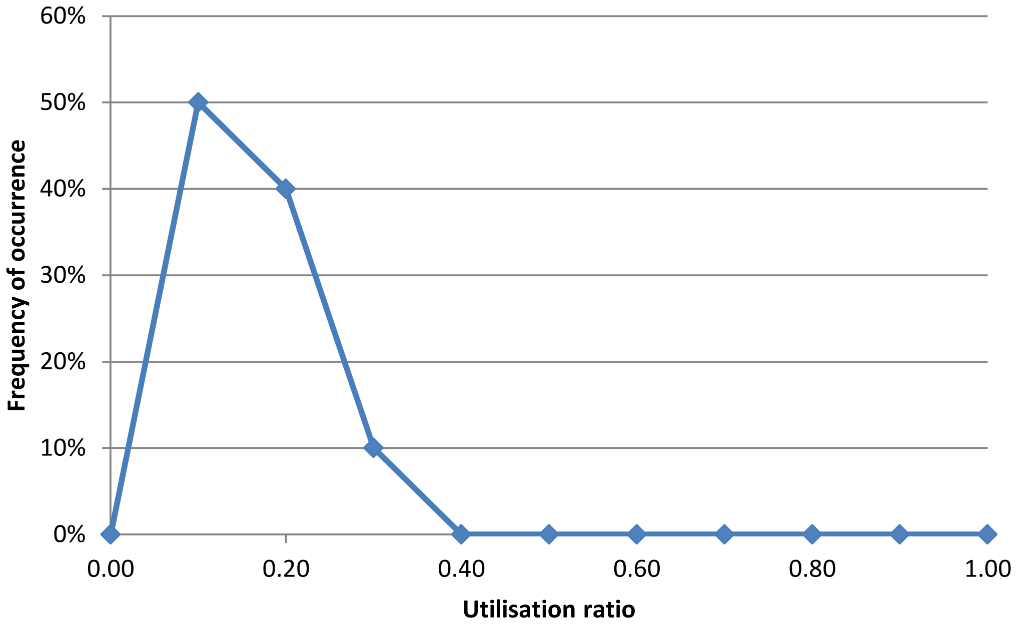

Figure 13: graph of frequency of occurrence against utilisation ratio for columns by floor and overall for building #3

Building #4

Type: school

62 of 62 beams analysed (100%)

Table 4: summary of results by floor for building #4

Level	No. beams analysed	% of total steel mass	Avg. U/R	Weighted avg. U/R	Top 5 Beams No.	Top 5 Beams %
Roof	29	78%	0.22	0.75	29	100%
1st floor	22	18%	0.16	0.23	22	100%
Other	*11*	*4%*	*0.06*	*0.05*	-	-
**TOTAL**	**62**	**100%**	**0.17**	**0.62**	**62**	**100%**

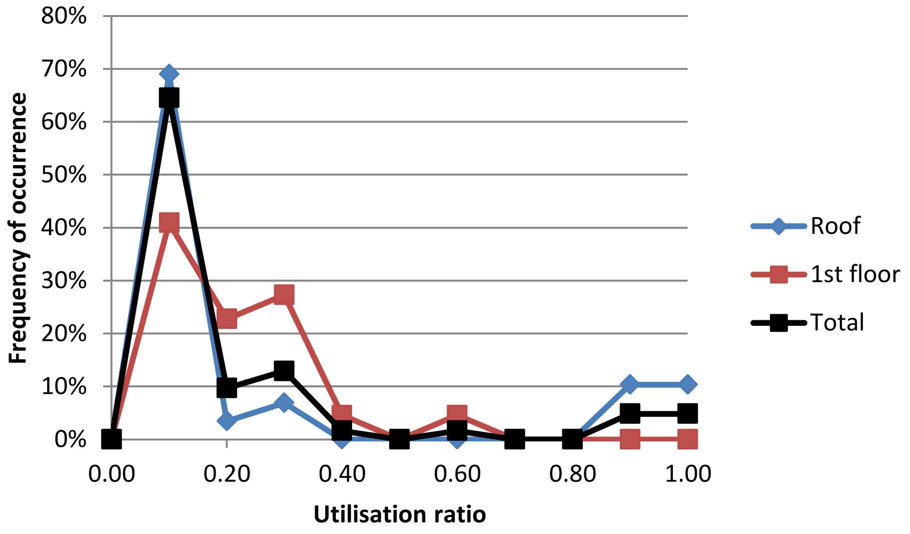

Figure 14: graph of frequency of occurrence against utilisation ratio for beams by floor and overall for building #4

1st floor

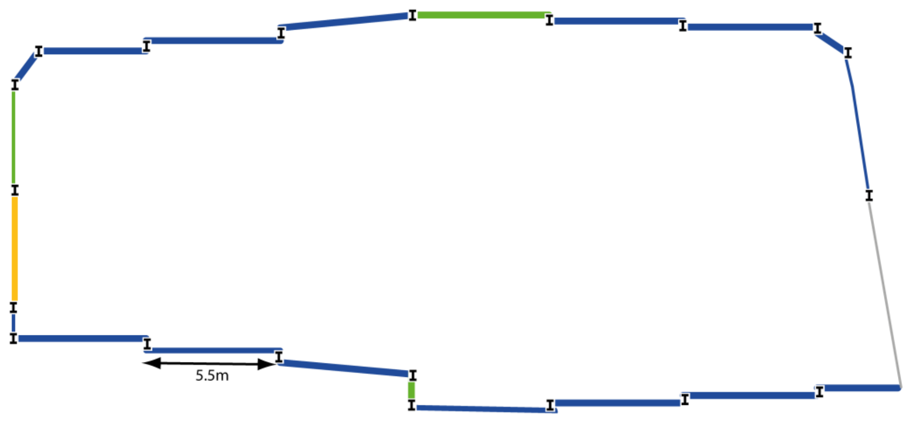

Figure 15: plot of floor showing beams coloured according to utilisation ratio (as per legend pg. S2)

Roof

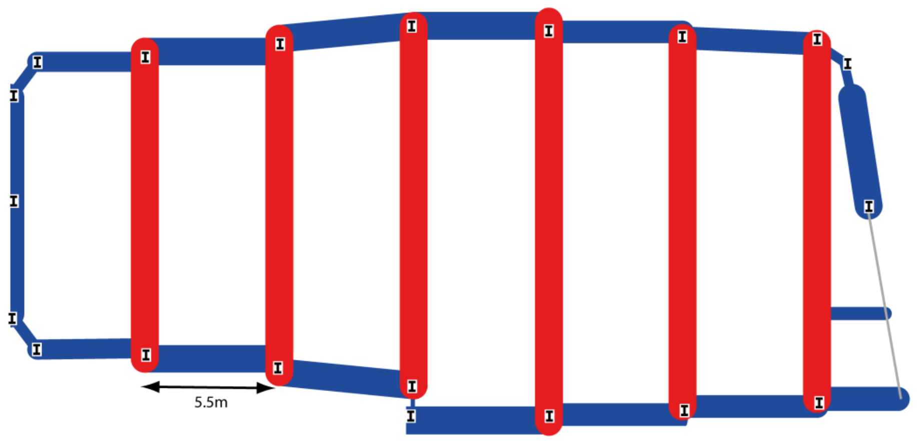

Figure 16: plot of floor showing beams coloured according to utilisation ratio (as per legend pg. S2)

Engineer's comments

Design deflection governed primarily; robustness not an issue.

Columns

21 of 21 columns analysed (100%)

Average U/R: 0.13

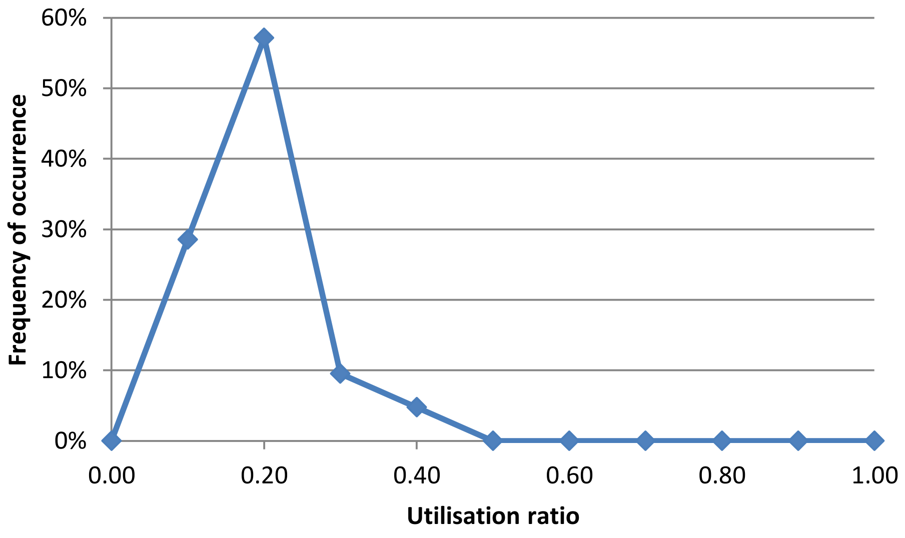

Figure 17: graph of frequency of occurrence against utilisation ratio for columns by floor and overall for building #4

Building #5

Type: office

21 of 21 beams analysed (100%)

Table 5: summary of results by floor for building #5

Level	No. beams analysed	% of total steel mass	Avg. U/R	Weighted avg. U/R	Top 5 Beams No.	%
Roof	21	100%	0.44	0.41	-	-

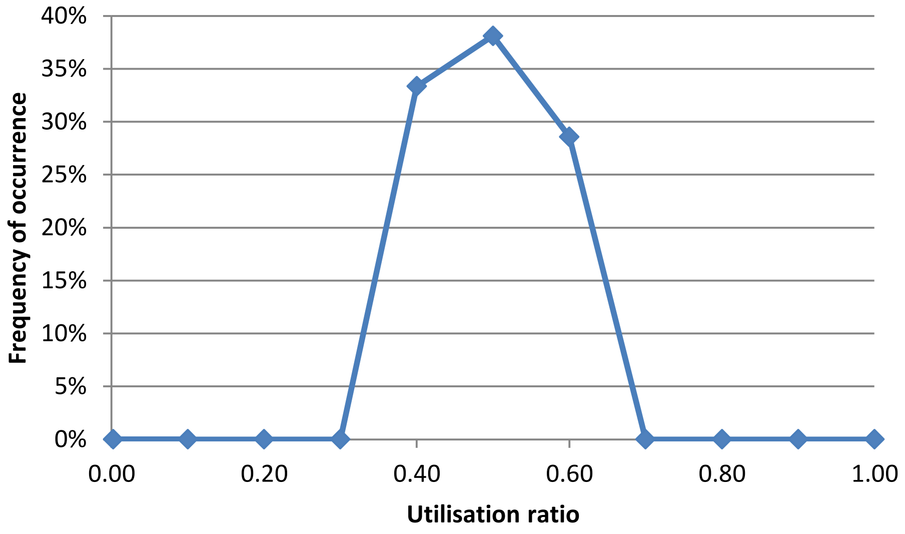

Figure 18: graph of frequency of occurrence against utilisation ratio for beams by floor and overall for building #5

Roof

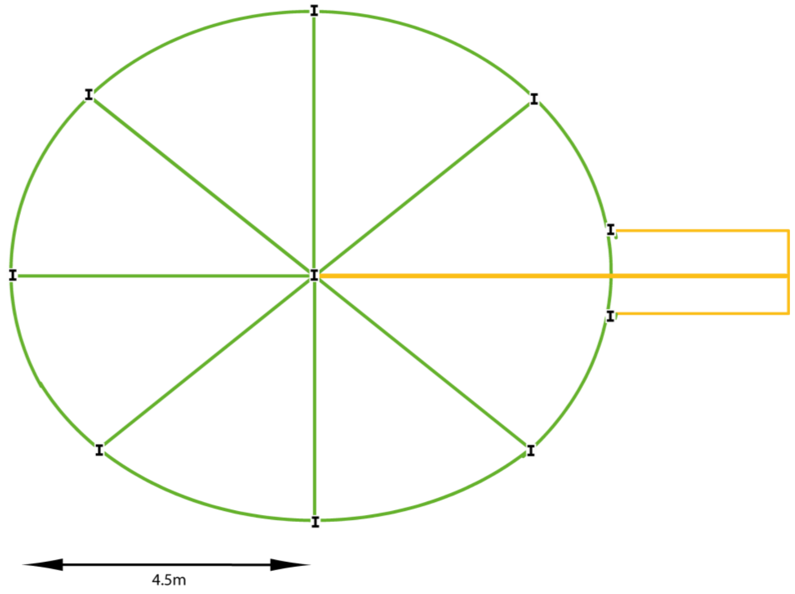

Figure 19: plot of floor showing beams coloured according to utilisation ratio (as per legend pg. S2)

Engineer's comments

The applied loads were reduced late in the project programme – too late to redesign, which resulted in spare capacity in places. Deflection governed most elements' design.

Columns

15 of 15 columns analysed (100%)

Average U/R: 0.64

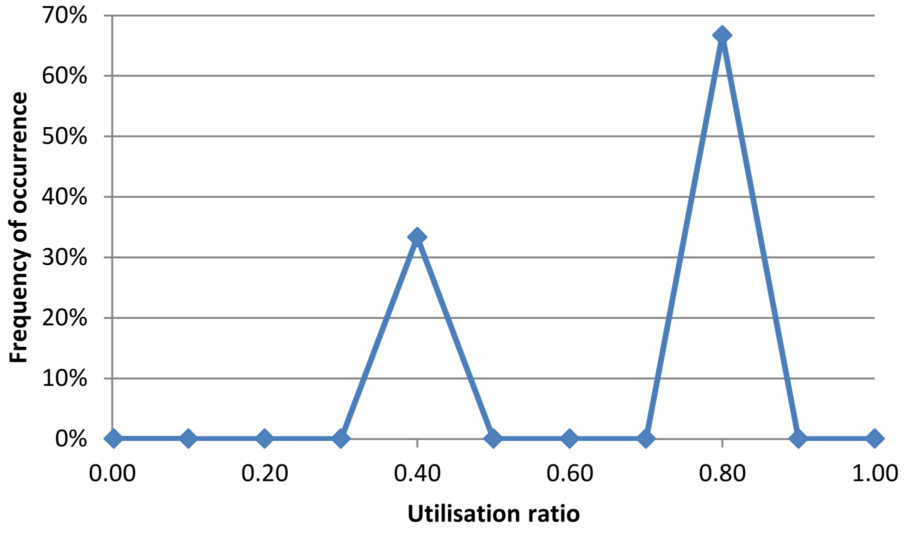

Figure 20: graph of frequency of occurrence against utilisation ratio for columns by floor and overall for building #5

Building #6

Type: office & education

700 of 1194 beams analysed (59%)

Table 6: summary of results by floor for building #6

Level	No. beams analysed	% of total steel mass	Avg. U/R	Weighted avg. U/R	Top 5 Beams No.	Top 5 Beams %
Roof	197	19%	0.12	0.22	139	71%
2nd floor	229	28%	0.11	0.27	195	85%
1st floor	197	34%	0.20	0.30	160	81%
Other	*77*	*19%*	*0.17*	*0.16*	-	-
**TOTAL**	**700**	**100%**	**0.15**	**0.25**	**541**	**77%**

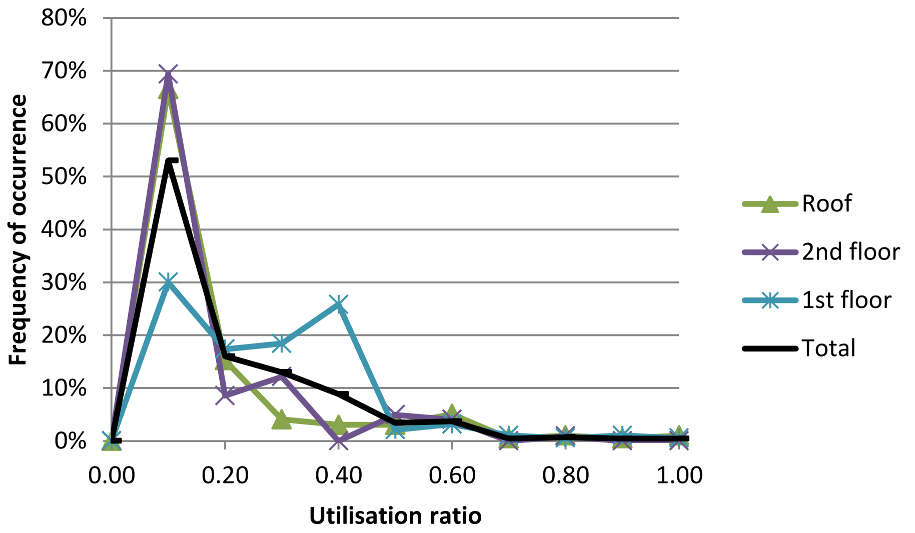

Figure 21: graph of frequency of occurrence against utilisation ratio for beams by floor and overall for building #6

1st floor

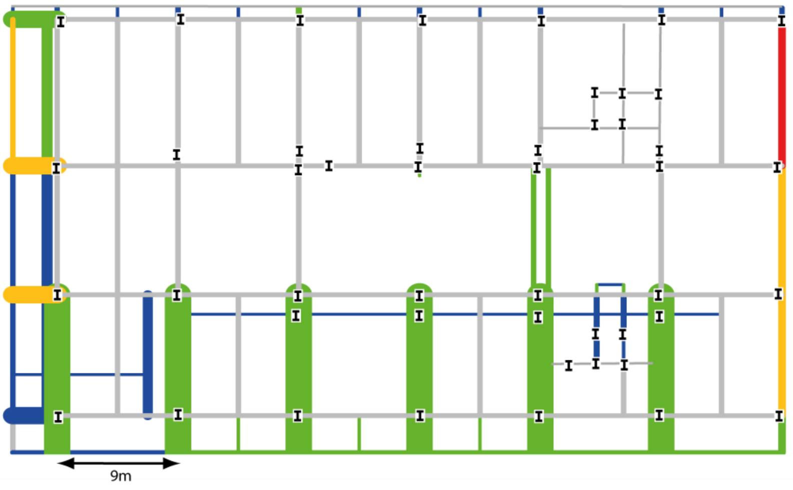

Figure 22: plot of floor showing beams coloured according to utilisation ratio (as per legend pg. S2)

2nd floor

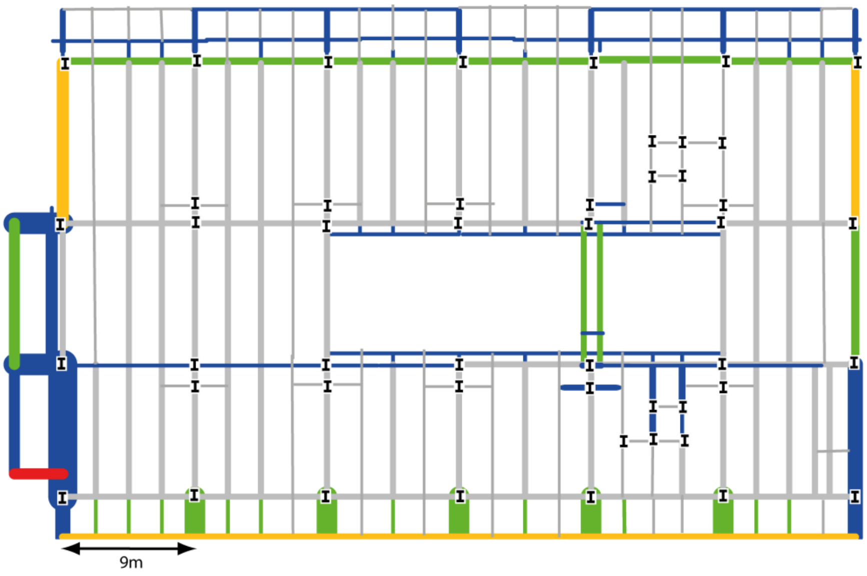

Figure 23: plot of floor showing beams coloured according to utilisation ratio (as per legend pg. S2)

Roof

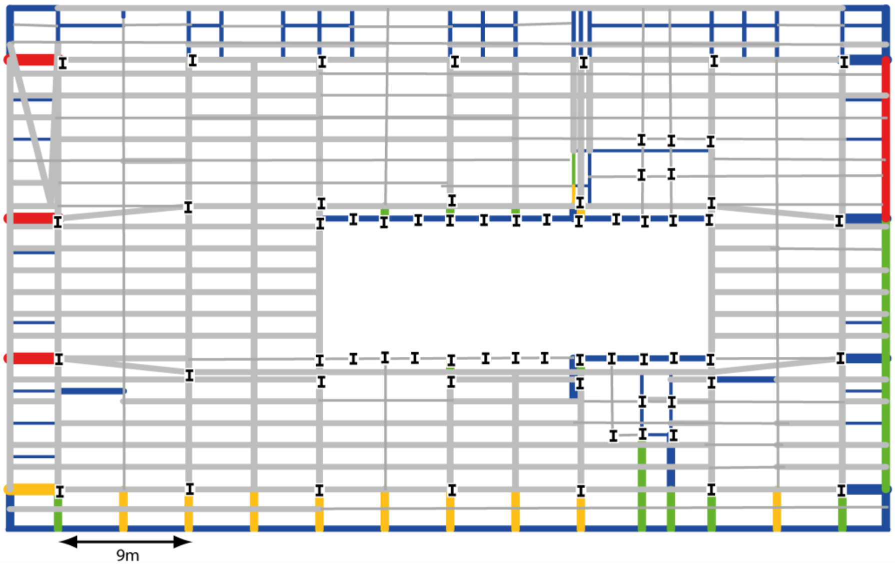

Figure 24: plot of floor showing beams coloured according to utilisation ratio (as per legend pg. S2)

Engineer’s comments

Computer model used mainly for stability and column design purposes – may explain why so many beams omitted from analysis. Design around edges governed either by vibration or by minimum sizes for façade supporting steelwork (to facilitate faster construction).

Columns

75 of 75 columns analysed (100%)

Average U/R: 0.42

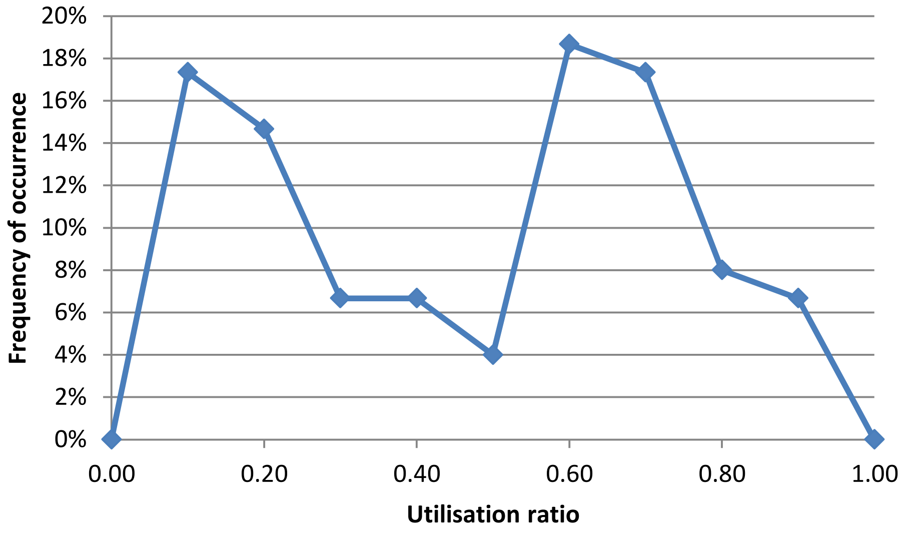

Figure 25: graph of frequency of occurrence against utilisation ratio for columns by floor and overall for building #6

Building #7

Type: school

766 of 908 beams analysed (84%)

Table 7: summary of results by floor for building #7

Level	No. beams analysed	% of total steel mass	Avg. U/R	Weighted avg. U/R	Top 5 Beams No.	Top 5 Beams %
Top Roof	125	8%	0.17	0.25	125	100%
Roof	196	23%	0.28	0.39	158	81%
3rd floor	114	20%	0.42	0.45	89	78%
2nd floor	129	21%	0.44	0.54	118	91%
1st floor	176	26%	0.40	0.53	150	86%
Other	*26*	*2%*	*0.21*	*0.14*	-	-
**Total**	**766**	**100%**	**0.33**	**0.45**	**474**	**62%**

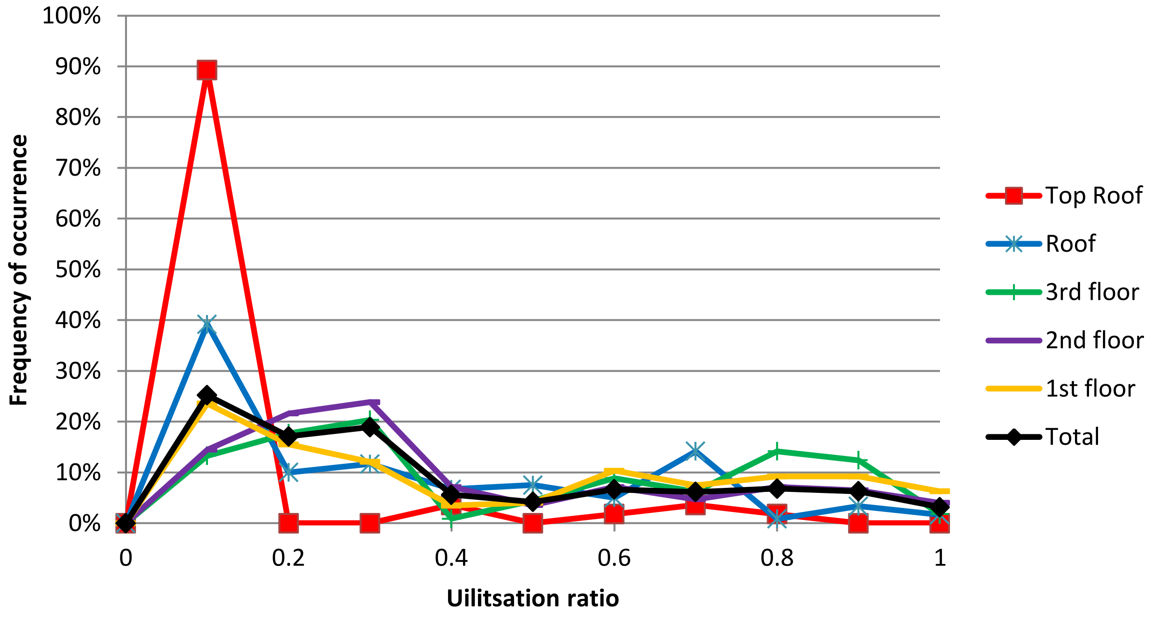

Figure 26: graph of frequency of occurrence against utilisation ratio for beams by floor and overall for building #7

1st floor

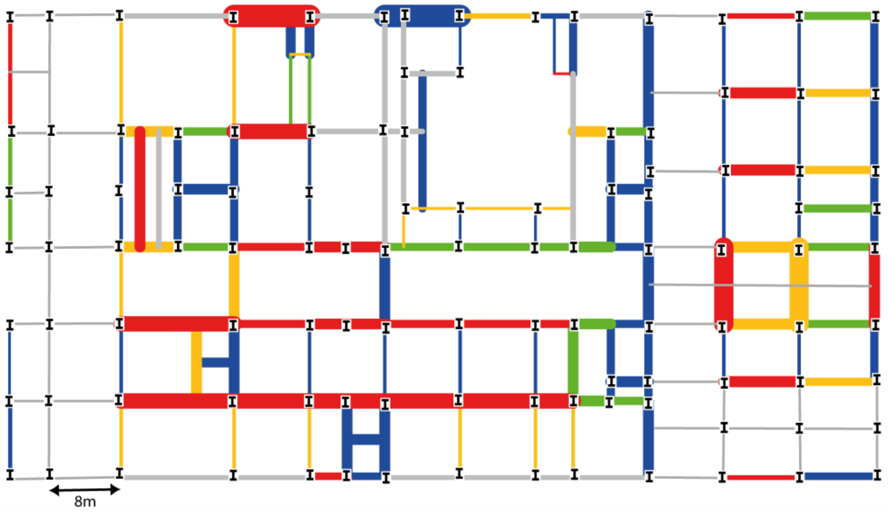

Figure 27: plot of floor showing beams coloured according to utilisation ratio (as per legend pg. S2)

2nd floor

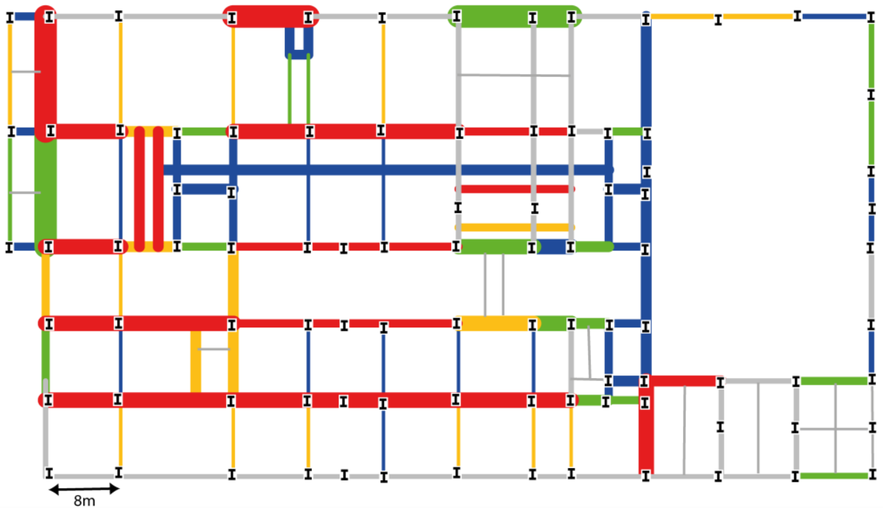

Figure 28: plot of floor showing beams coloured according to utilisation ratio (as per legend pg. S2)

3rd floor

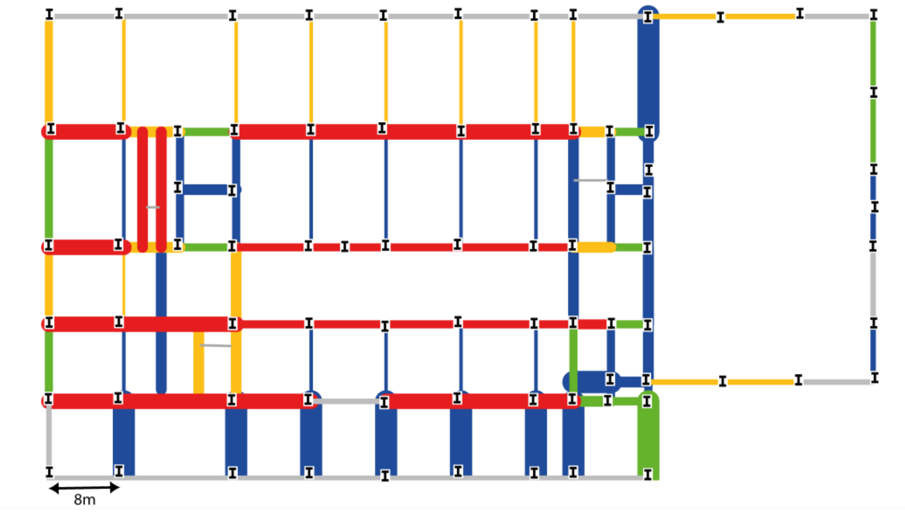

Figure 29: plot of floor showing beams coloured according to utilisation ratio (as per legend pg. S2)

Roof

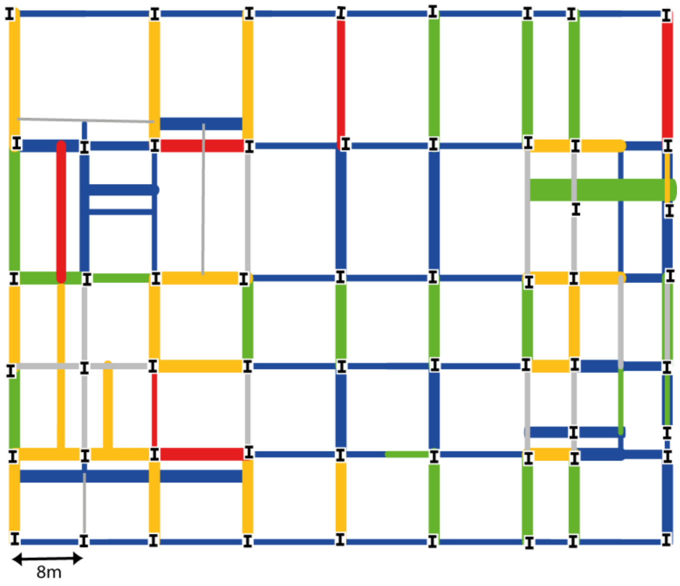

Figure 30: plot of floor showing beams coloured according to utilisation ratio (as per legend pg. S2)

There was not sufficient information to plot the Top Roof level.

Engineer's comments

Vibration governed in some places but mainly stress and deflection governed.

Columns

103 of 113 columns analysed (91%)

Average U/R: 0.47

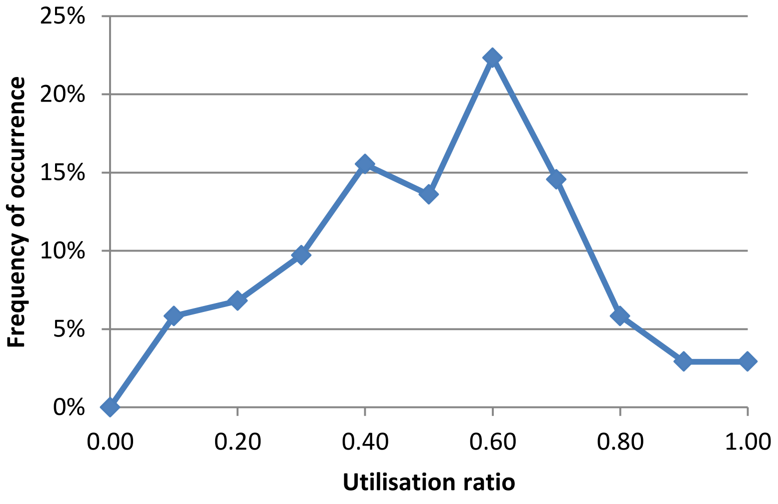

Figure 31: graph of frequency of occurrence against utilisation ratio for columns by floor and overall for building #7

Building #8

Type: office

375 of 519 beams analysed (72%)

Table 8: summary of results by floor for building #8

Level	No. beams analysed	% of total steel mass	Avg. U/R	Weighted avg. U/R	Top 5 Beams No.	Top 5 Beams %
Roof	101	23%	0.23	0.30	83	82%
4th floor	70	18%	0.34	0.43	63	90%
3rd floor	67	16%	0.33	0.41	67	100%
2nd floor	70	18%	0.34	0.43	63	90%
1st floor	66	18%	0.35	0.44	57	86%
**TOTAL**	**375**	**100%**	**0.31**	**0.39**	**312**	**83%**

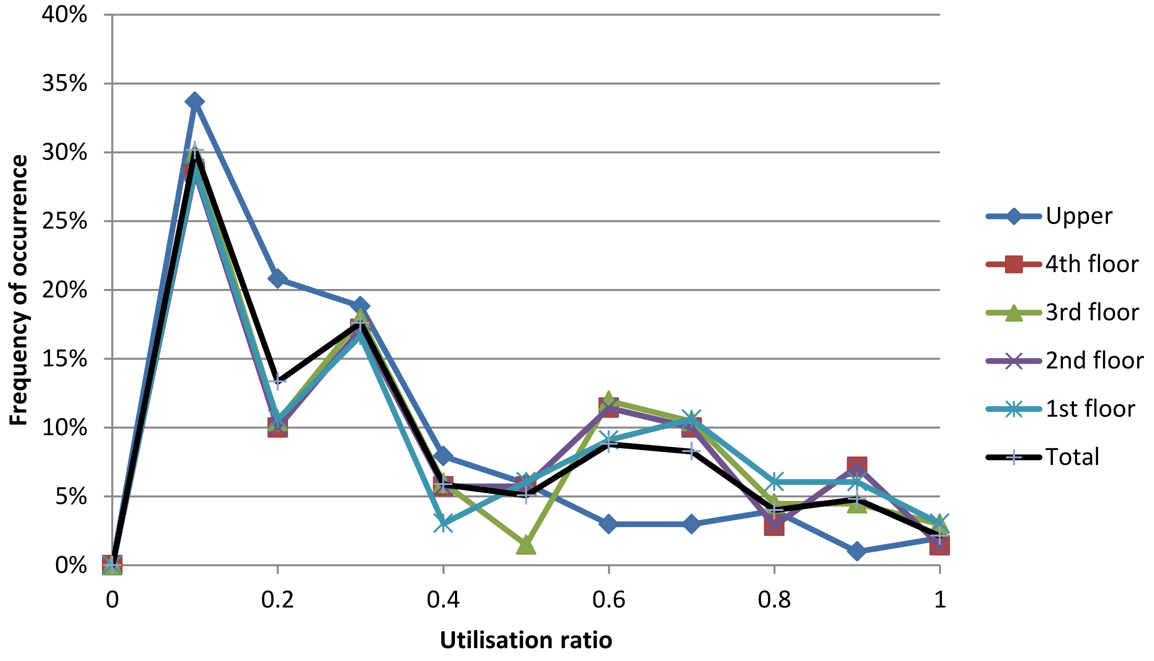

Figure 32: graph of frequency of occurrence against utilisation ratio for beams by floor and in total for building #8

No data on beam layout was available for this building; therefore floor plots could not be created.

Engineer's comments

Most of the beams not analysed in model were specially-fabricated beams. These beams are expected to have high U/R.

Columns

38 of 40 columns analysed (95%)

Average U/R: 0.72

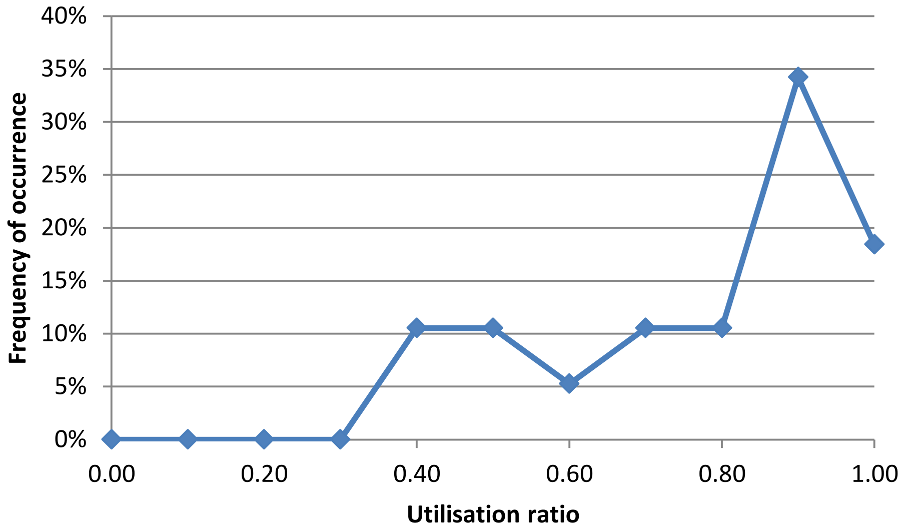

Figure 33: graph of frequency of occurrence against utilisation ratio for columns by floor and overall for building #8

Building #9

Type: office

512 of 606 beams analysed (84%)

Table 9: summary of results by floor for building #9

Level	No. beams analysed	% of total steel mass	Avg. U/R	Weighted avg. U/R	Top 5 Beams No.	Top 5 Beams %
Roof	131	27%	0.34	0.46	80	61%
3rd floor	112	24%	0.44	0.55	68	61%
2nd floor	115	25%	0.43	0.54	70	61%
1st floor	111	22%	0.38	0.47	64	58%
Other	*43*	*3%*	*0.09*	*0.19*	-	-
**TOTAL**	**512**	**100%**	**0.37**	**0.50**	**294**	**57%**

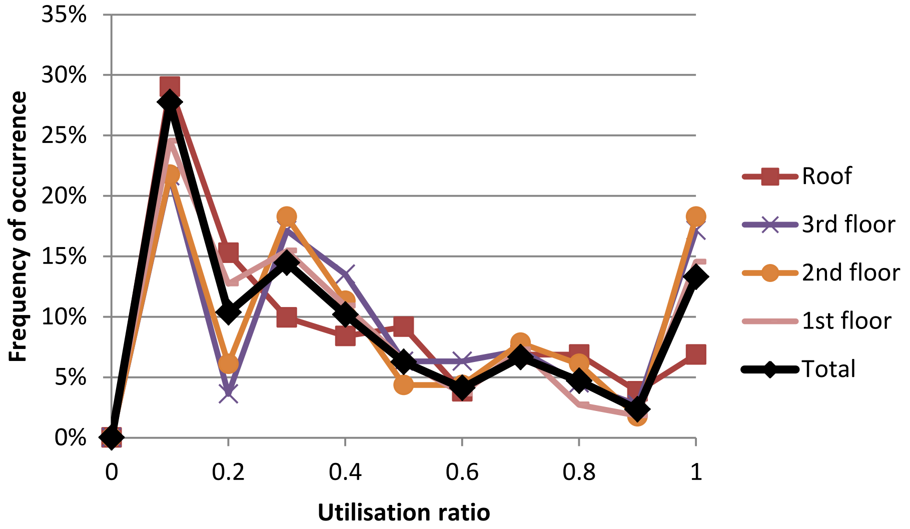

Figure 34: graph of frequency of occurrence against utilisation ratio for beams by floor and overall for building #9

No data on beam layout was available for this building; therefore floor plots could not be created.

Engineer's comments

Vibration was a governing criterion in a small area. Many of the beams not analysed were specially-fabricated beams, expected to have high U/R.

Columns

56 of 59 columns analysed (95%)

Average U/R: 0.60

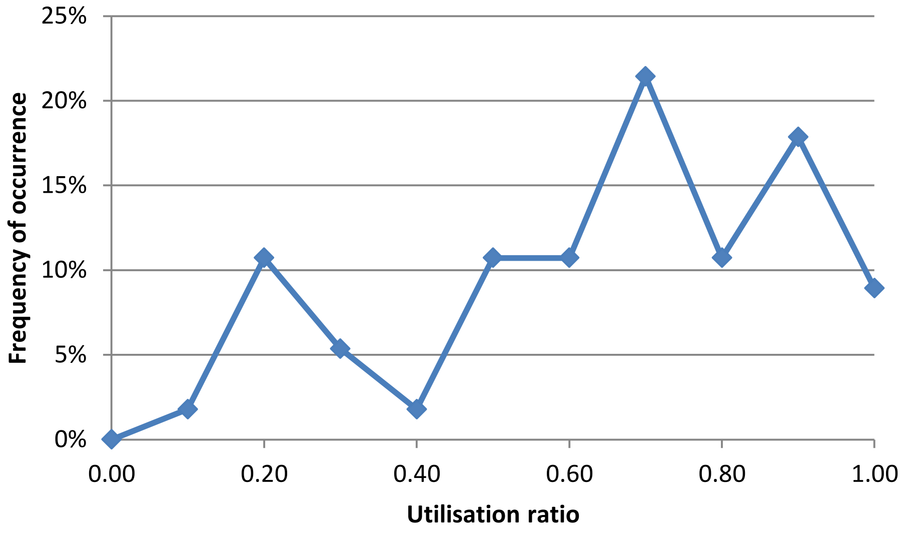

Figure 35: graph of frequency of occurrence against utilisation ratio for columns by floor and overall for building #9

Building #10

Type: office

35 of 48 beams analysed (73%)

Table 10: summary of results by floor for building #10

Level	No. beams analysed	% of total steel mass	Avg. U/R	Weighted avg. U/R	Top 5 Beams No.	%
Roof	35	100%	0.90	0.96	3	100

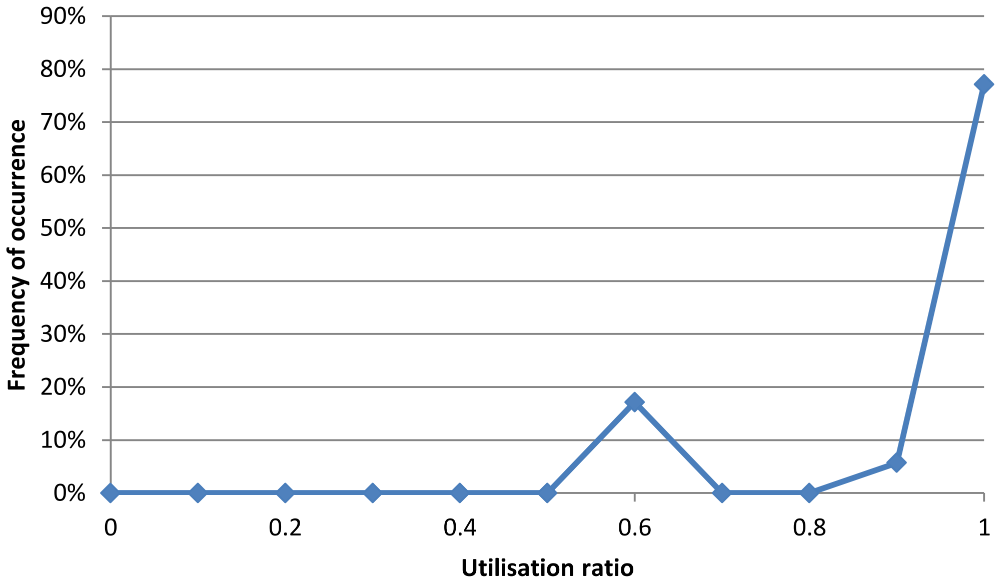

Figure 36: graph of frequency of occurrence against utilisation ratio for beams by floor and overall for building #10

Roof

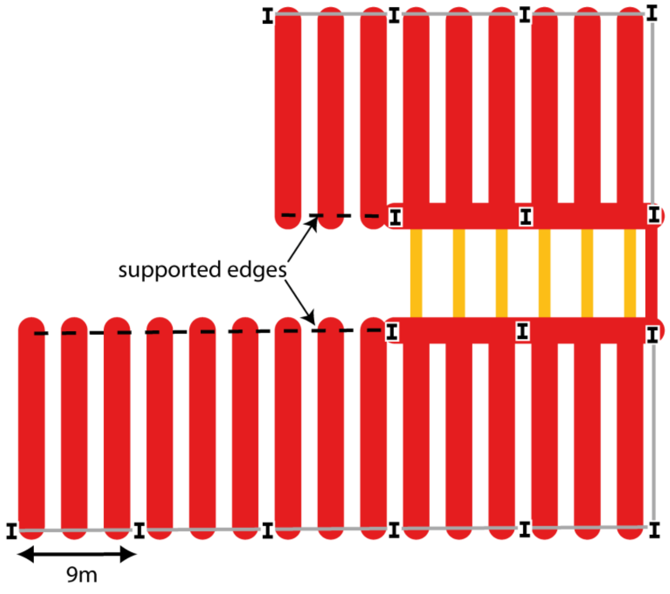

Figure 37: plot of floor showing beams coloured according to utilisation ratio (as per legend pg. S2)

Engineer’s comments:

Deflections governed design. Not surprised that had high U/R as had time to design thoroughly and no late changes were made.

Insufficient information was available about the columns in this building to allow analysis.

Building #11

Type: school

379 of 503 beams analysed (75%)

Table 11: summary of results by floor for building #11

Level	No. beams analysed	% of total steel mass	Avg. U/R	Weighted avg. U/R	Top 5 Beams No.	Top 5 Beams %
Roof	98	24%	0.66	0.70	74	76%
2nd floor	143	39%	0.64	0.68	108	77%
1st floor	138	24%	0.63	0.68	106	78%
**TOTAL**	**379**	**100%**	**0.64**	**0.68**	**269**	**71%**

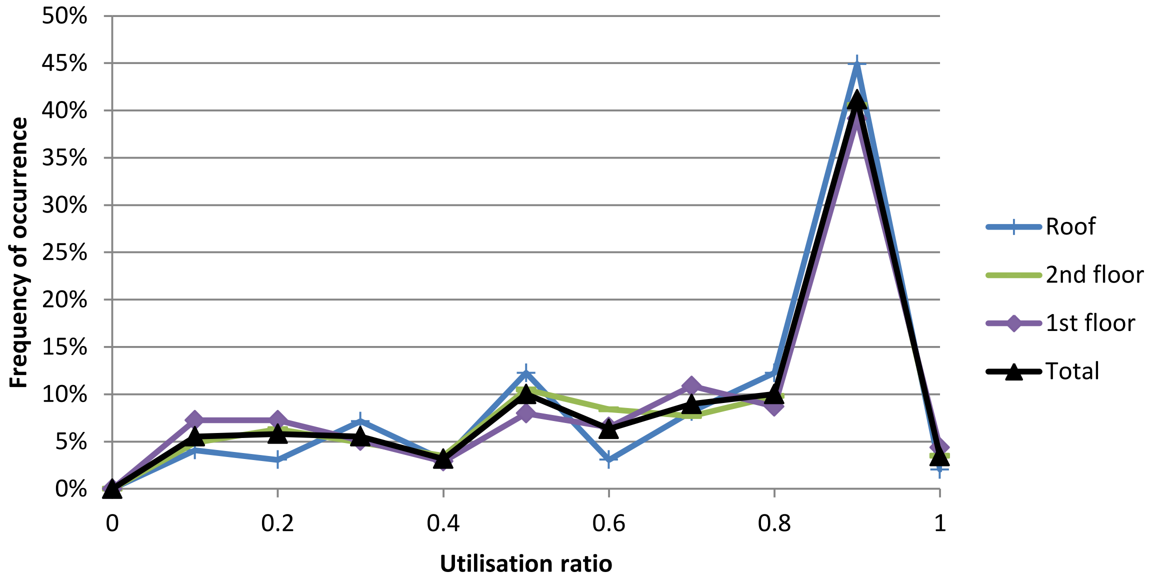

Figure 38: graph of frequency of occurrence against utilisation ratio for beams by floor and overall for building #11

Insufficient data on beam layout were available for this building to create layout plots.

Engineer's comments

Steelwork was rationalised to enable cheaper procurement – fabricator further rationalised the design also.

Columns

55 of 109 columns analysed (50%)

Average U/R: 0.69

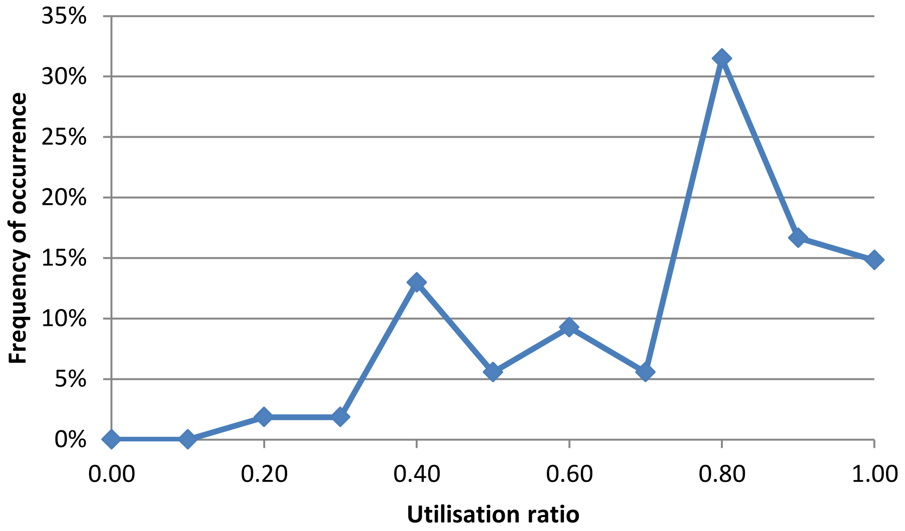

Figure 39: graph of frequency of occurrence against utilisation ratio for columns by floor and overall for building #11

Building #12

Type: school

526 of 578 beams analysed (91%)

Table 12: summary of results by floor for building #12

Level	No. beams analysed	% of total steel mass	Avg. U/R	Weighted avg. U/R	Top 5 Beams No.	Top 5 Beams %
2nd floor	241	42%	0.41	0.57	178	74%
1st floor	201	42%	0.61	0.69	93	46%
Other	*84*	*16%*	*0.30*	*0.44*	-	-
**TOTAL**	**526**	**100%**	**0.47**	**0.60**	339	64%

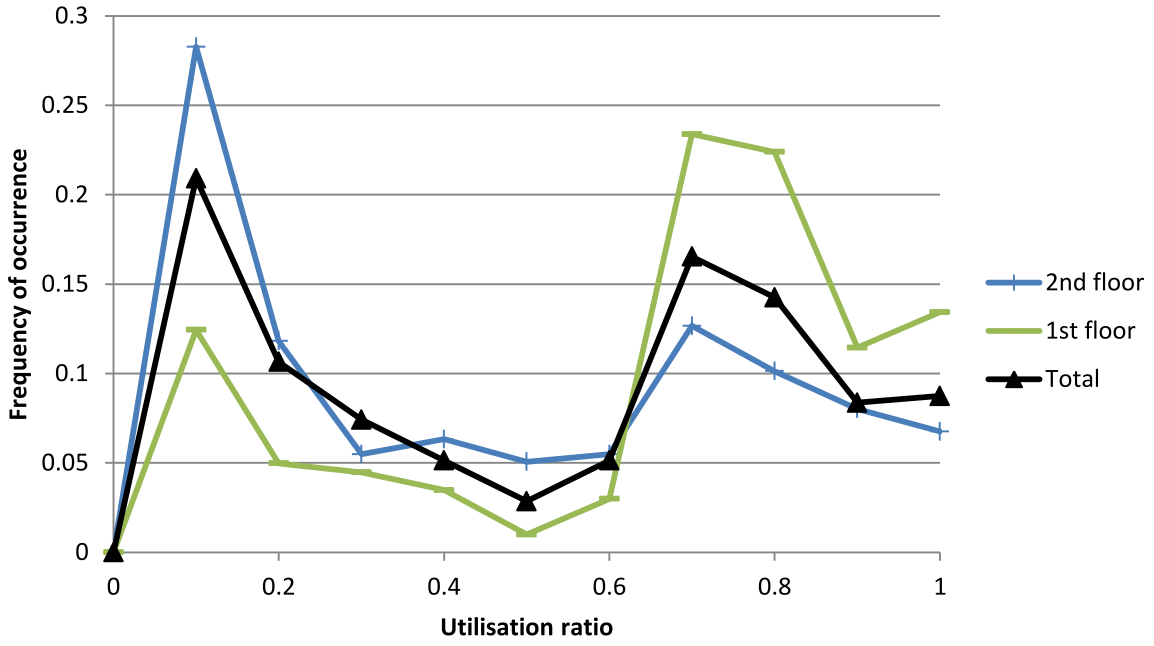

Figure 40: graph of frequency of occurrence against utilisation ratio for beams by floor and overall for building #12

1st floor

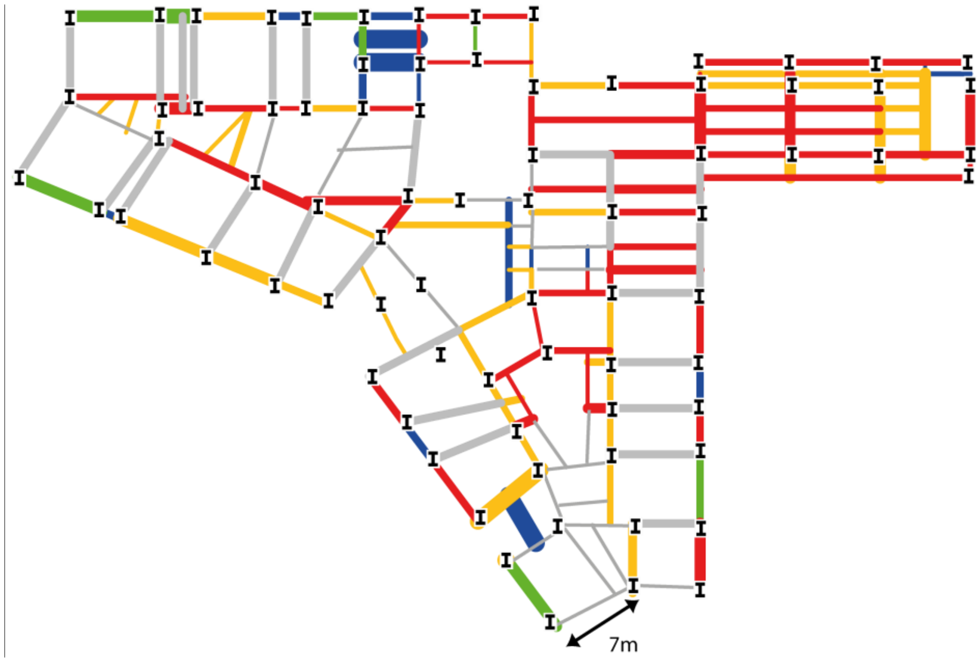

Figure 41: plot of floor showing beams coloured according to utilisation ratio (as per legend pg. S2)

2nd floor

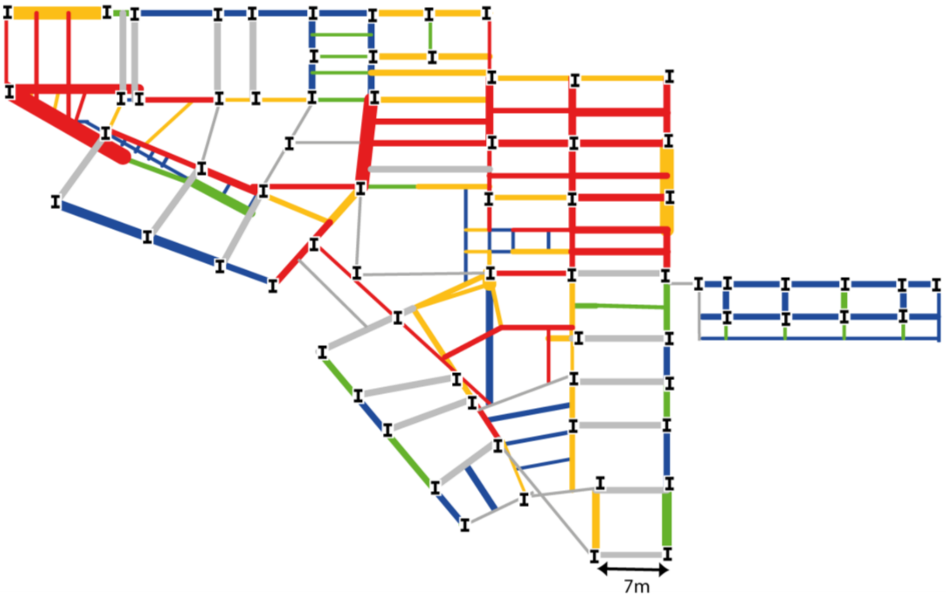

Figure 42: plot of floor showing beams coloured according to utilisation ratio (as per legend pg. S2)

Engineer's comments

Steelwork was rationalised to enable cheaper procurement – fabricator further rationalised the design also. Regular column grid prevented by client desire to provide minimum required area (lower heating costs) and to minimise cladding cost.

Columns

100 of 108 columns analysed (93%)

Average U/R: 0.49

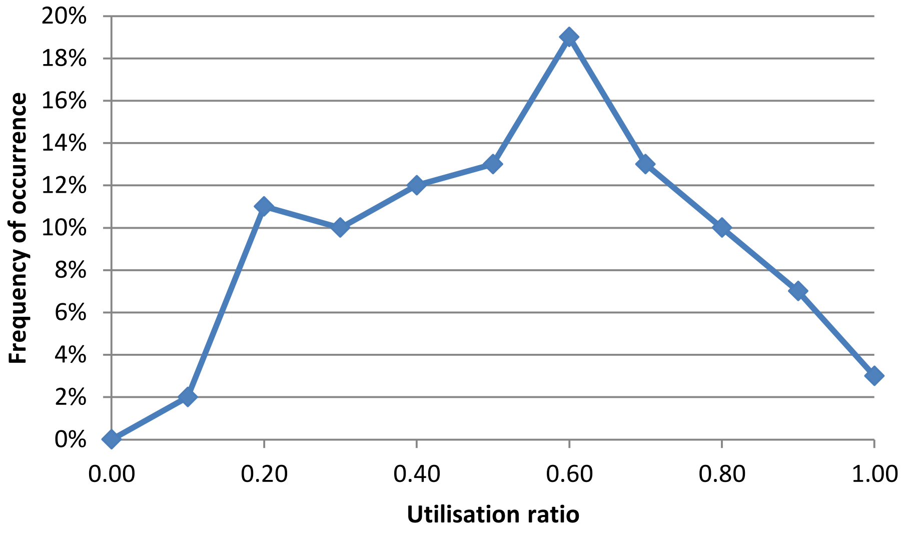

Figure 43: graph of frequency of occurrence against utilisation ratio for columns by floor and overall for building #12

Building #13

Type: school

311 of 372 beams analysed (84%)

Table 13: summary of results by floor for building #13

Level	No. beams analysed	% of total steel mass	Avg. U/R	Weighted avg. U/R	Top 5 Beams No.	Top 5 Beams %
1st floor	86	18%	0.47	0.64	79	92%
Other	*225*	*82%*	*0.36*	*0.46*	-	-
**TOTAL**	**311**	**100%**	**0.39**	**0.49**	**230**	**75%**

This building was composed of many different levels with less than 20 beams on each, which did not merit plotting individually, hence only the first floor is examined in detail.

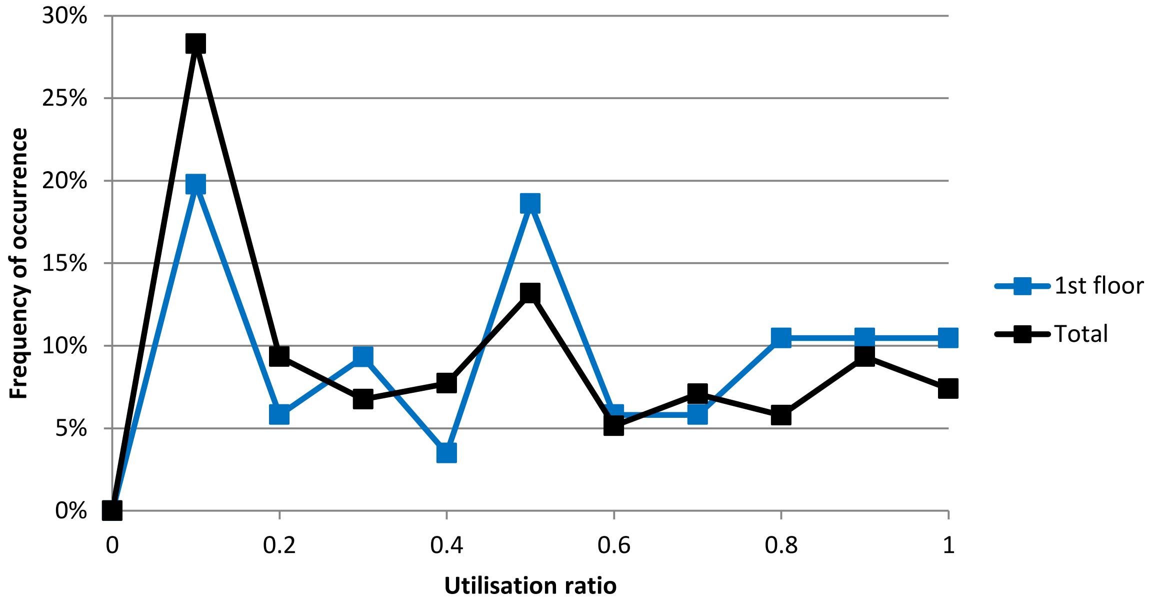

Figure 44: graph of frequency of occurrence against utilisation ratio for beams by floor and overall for building #13

1st floor

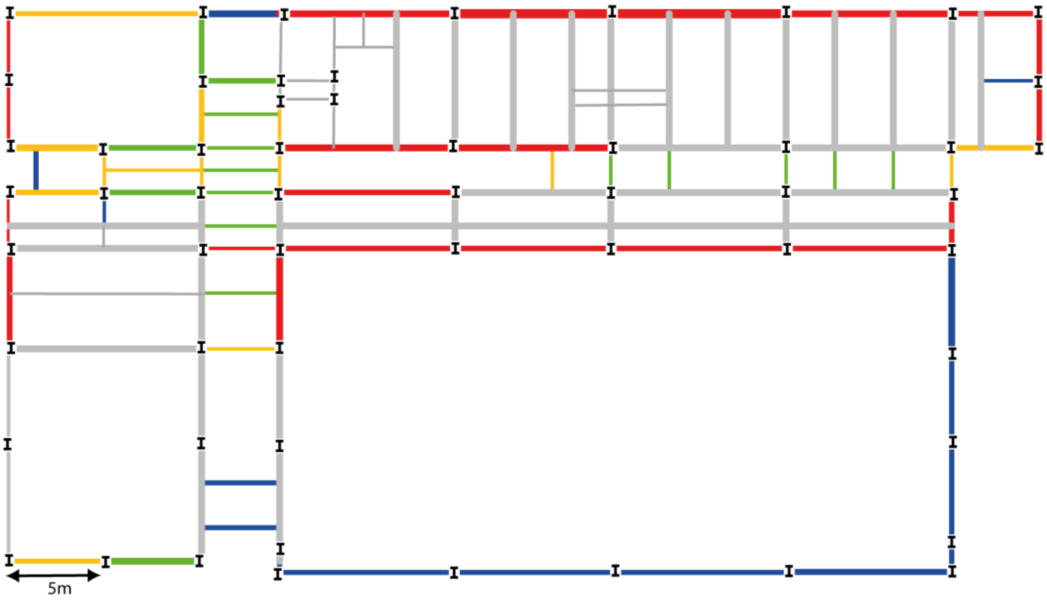

Figure 45: plot of floor showing beams coloured according to utilisation ratio (as per legend pg. S2)

Engineer’s comments

Steelwork was rationalised to enable cheaper procurement – fabricator further rationalised the design also.

Columns

64 of 74 columns analysed (86%)

Average U/R: 0.52

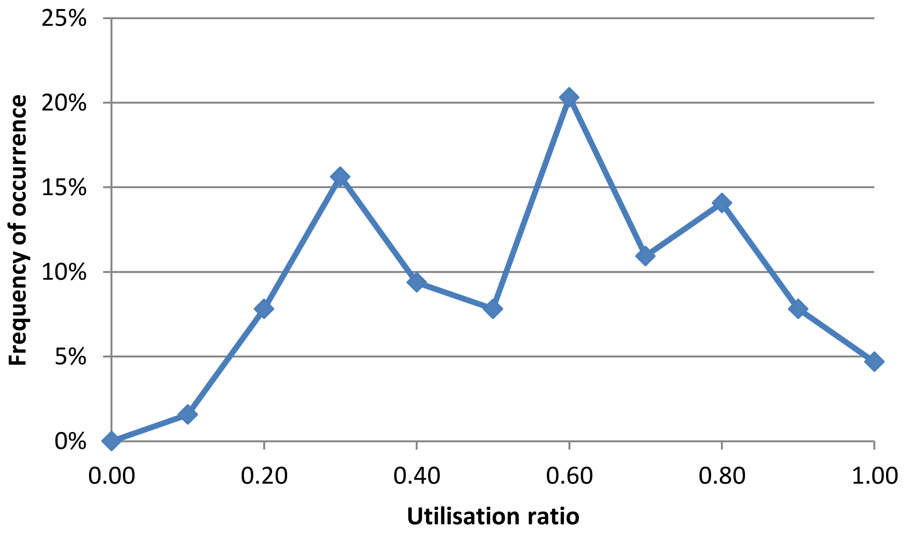

Figure 46: graph of frequency of occurrence against utilisation ratio for columns by floor and overall for building #13

Building #14

Type: school

751 of 760 beams analysed (99%)

Table 14: summary of results by floor for building #14

Level	No. beams analysed	% of total steel mass	Avg. U/R	Weighted avg. U/R	Top 5 Beams No.	Top 5 Beams %
Roof	86	11%	0.24	0.42	65	79%
2nd floor	330	52%	0.23	0.33	275	84%
1st floor	322	35%	0.29	0.44	241	75%
Other	*13*	*2%*	*0.23*	*0.41*	-	-
**TOTAL**	**751**	**100%**	**0.26**	**0.38**	**585**	**78%**

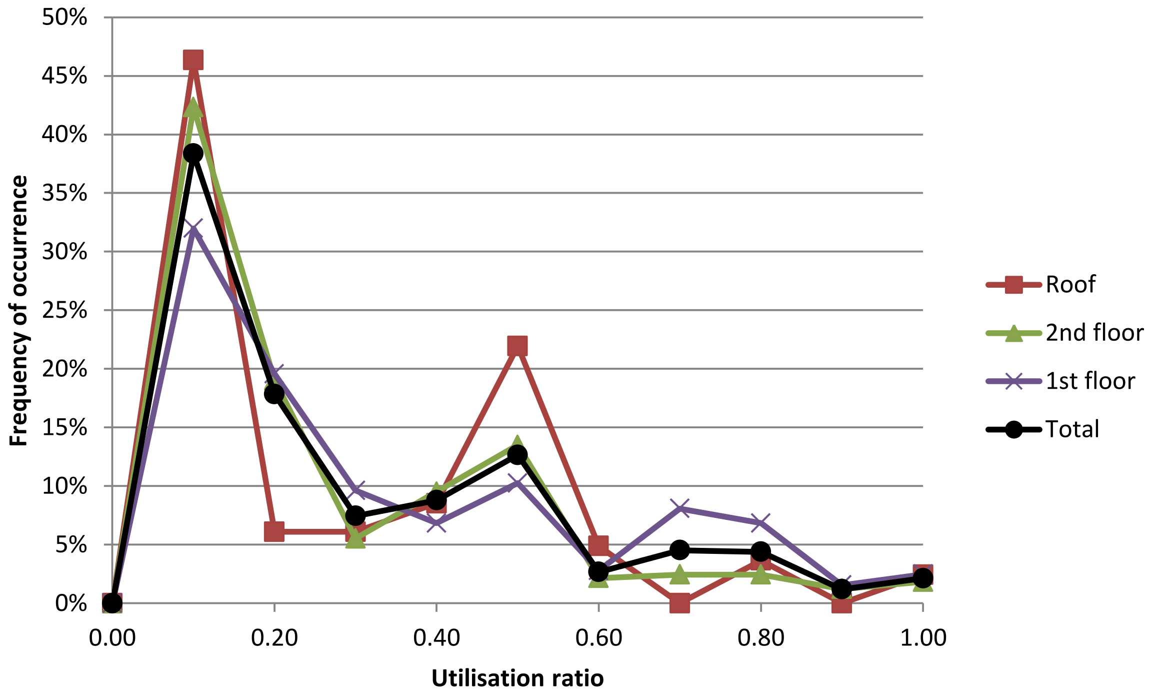

Figure 47: graph of frequency of occurrence against utilisation ratio for beams by floor and overall for building #14

1st floor

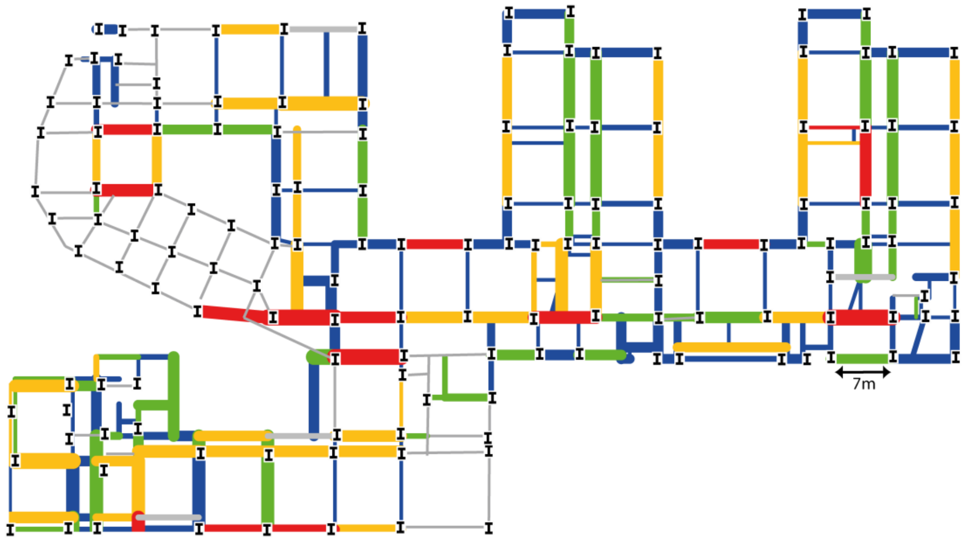

Figure 48: plot of floor showing beams coloured according to utilisation ratio (as per legend pg. S2)

2nd floor

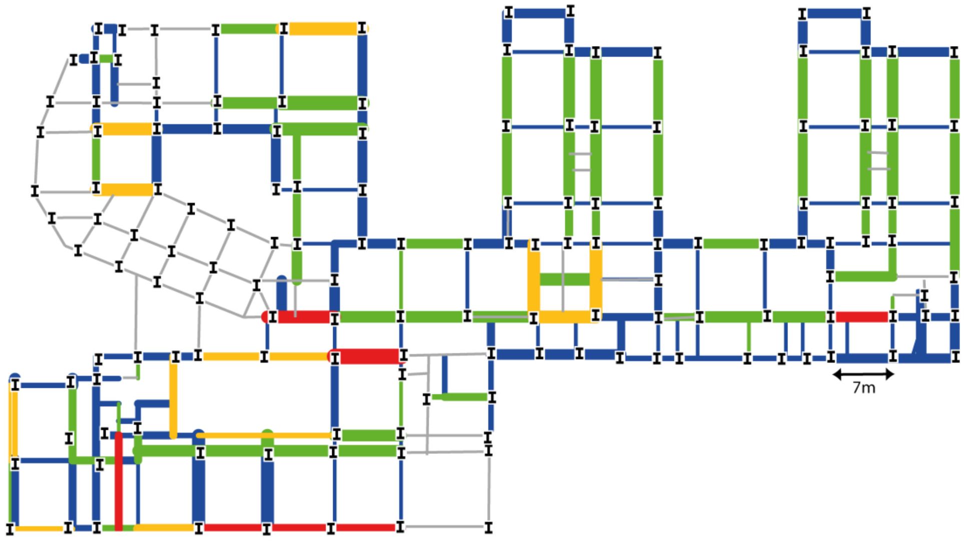

Figure 49: plot of floor showing beams coloured according to utilisation ratio (as per legend pg. S2)

Roof

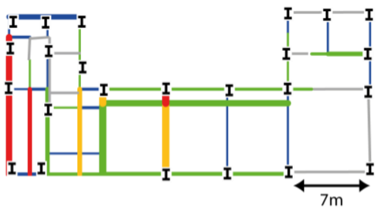

Figure 50: plot of floor showing beams coloured according to utilisation ratio (as per legend pg. S2)

Roof column layout drawings were not available so Figure 53 was plotted using engineering intuition based on the other two floors.

Engineer’s comments

Many beams governed by loading during construction. Increasing mass to take this load was deemed the cheapest option, as other solutions required more labour on site. A small number of beams were governed by vibration concerns.

Columns

166 of 168 columns analysed (99%)

Average U/R: 0.54

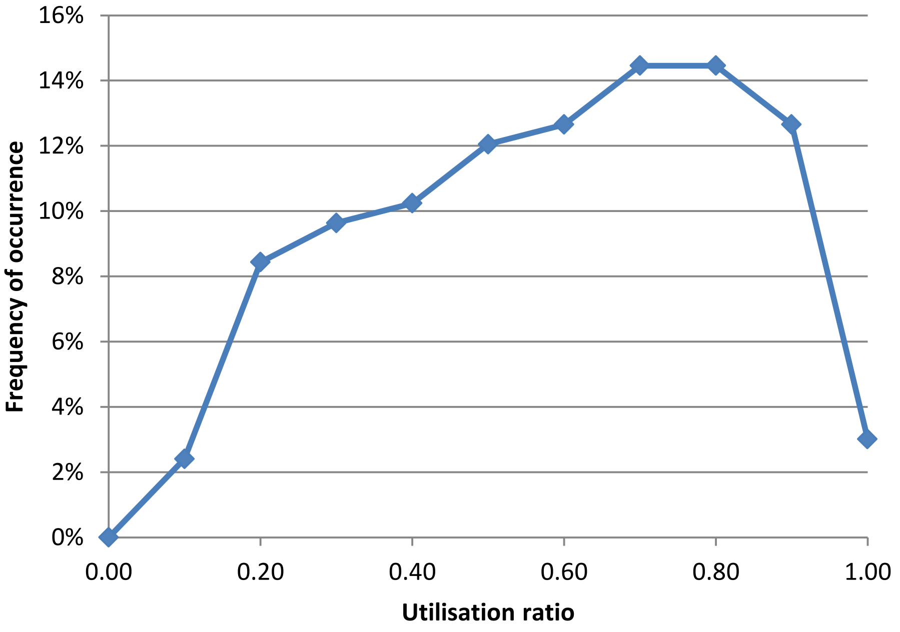

Figure 51: graph of frequency of occurrence against utilisation ratio for columns by floor and overall for building #14

Building #15

Type: mixed-use residential and retail

1447 of 2230 beams analysed (65%)

Table 15: summary of results by floor for building #15

Level	No. beams analysed	% of total steel mass	Avg. U/R	Weighted avg. U/R	Top 5 Beams No.	Top 5 Beams %
Roofs	75	4%	0.17	0.31	59	79%
7th – 11th floor*	64	3%	0.10	0.15	55	86%
6th floor	105	7%	0.19	0.40	87	83%
3rd - 5th floor*	154	8%	0.16	0.35	129	84%
2nd floor	154	8%	0.16	0.35	100	65%
1st floor	127	9%	0.24	0.46	126	99%
Ground floor	115	17%	0.29	0.48	82	71%
Basement	89	16%	0.32	0.46	77	87%
**TOTAL**	**1447**	**100%**	**0.18**	**0.37**	**1073**	**74%**

*Floors 3-5 have very similar beam numbers, sections, utilisations and layouts so the data for just one of these floors is presented in Table 15, similarly for floors 7-11.

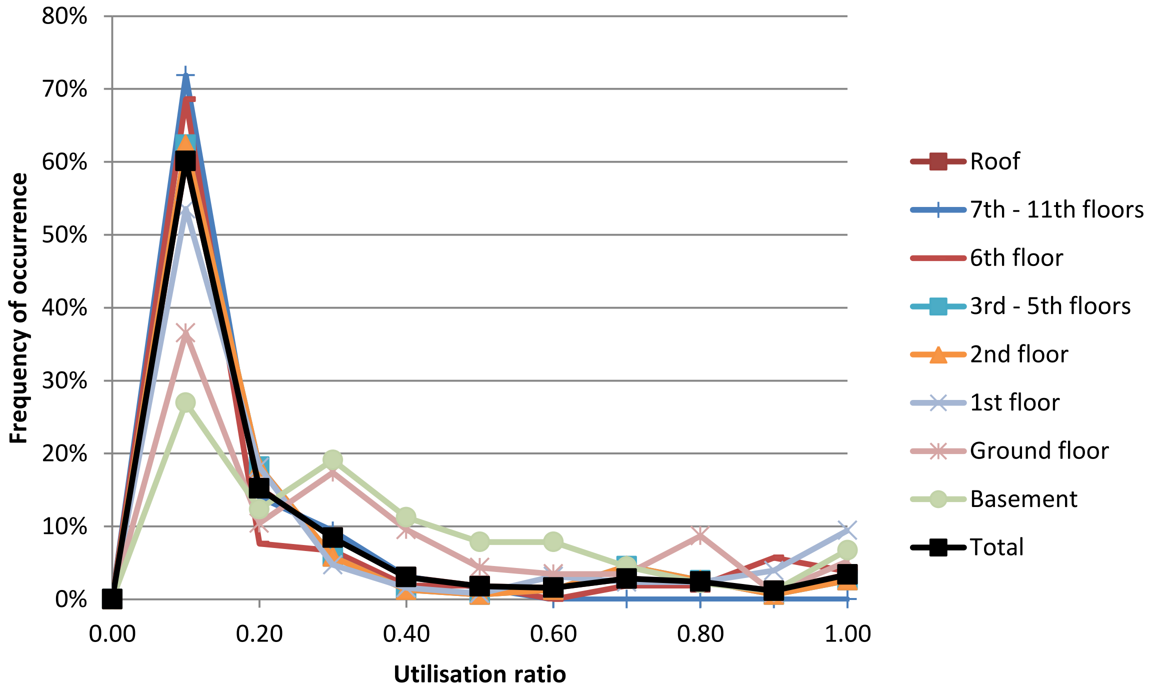

Figure 52: graph of frequency of occurrence against utilisation ratio for beams by floor and overall for building #15

Basement

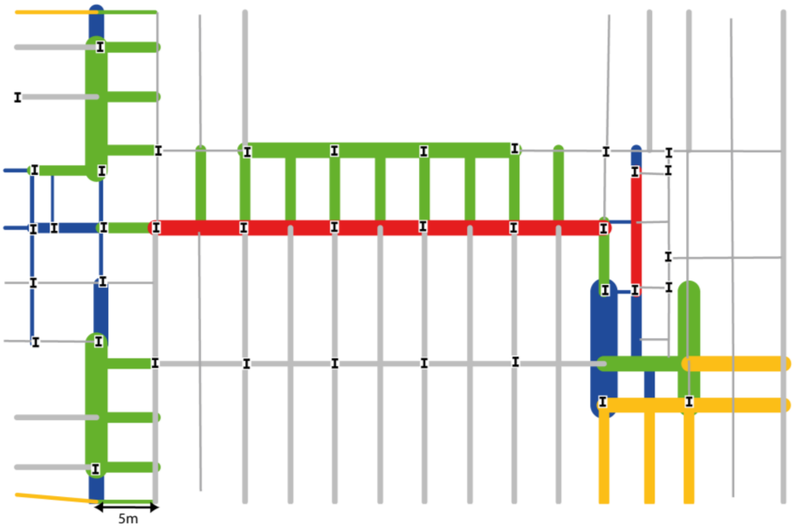

Figure 53: plot of floor showing beams coloured according to utilisation ratio (as per legend pg. S2)

Ground floor

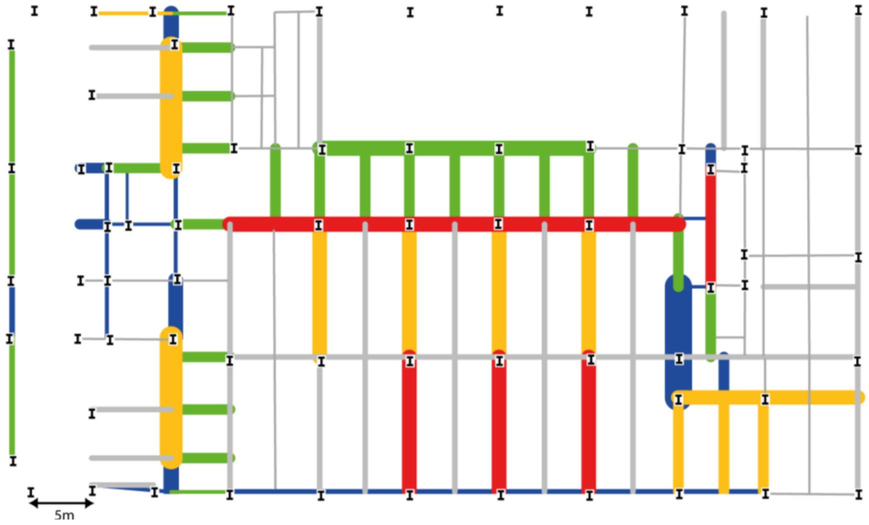

Figure 54: plot of floor showing beams coloured according to utilisation ratio (as per legend pg. S2)

1st floor

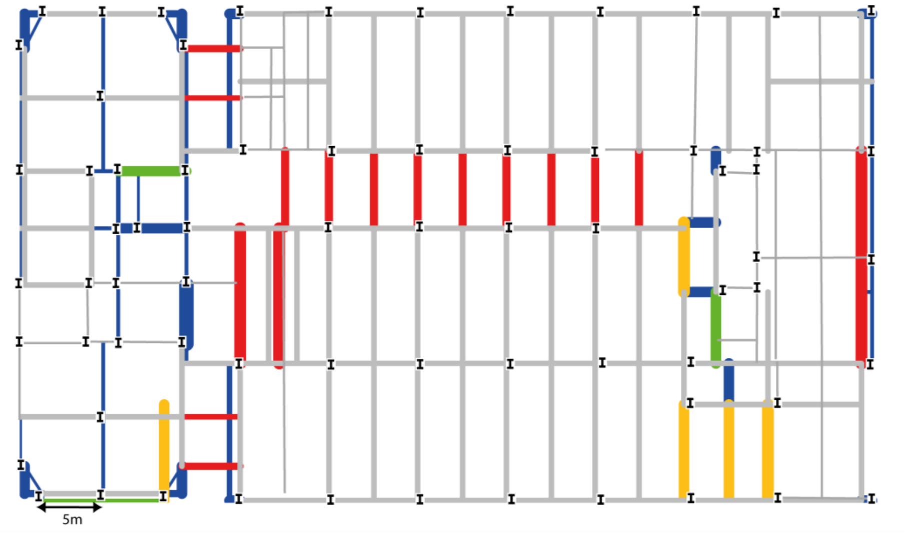

Figure 55: plot of floor showing beams coloured according to utilisation ratio (as per legend pg. S2)

2nd floor

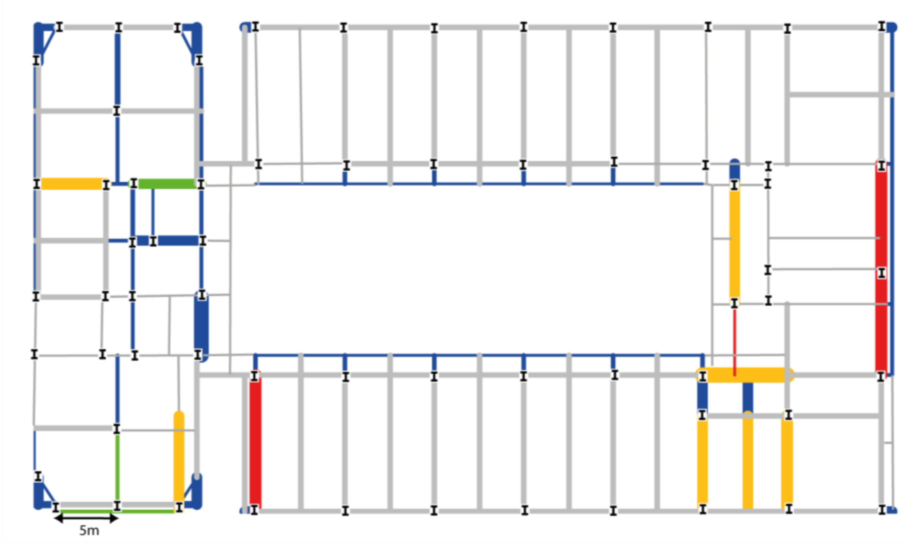

Figure 56: plot of floor showing beams coloured according to utilisation ratio (as per legend pg. S2)

3rd – 5th floors

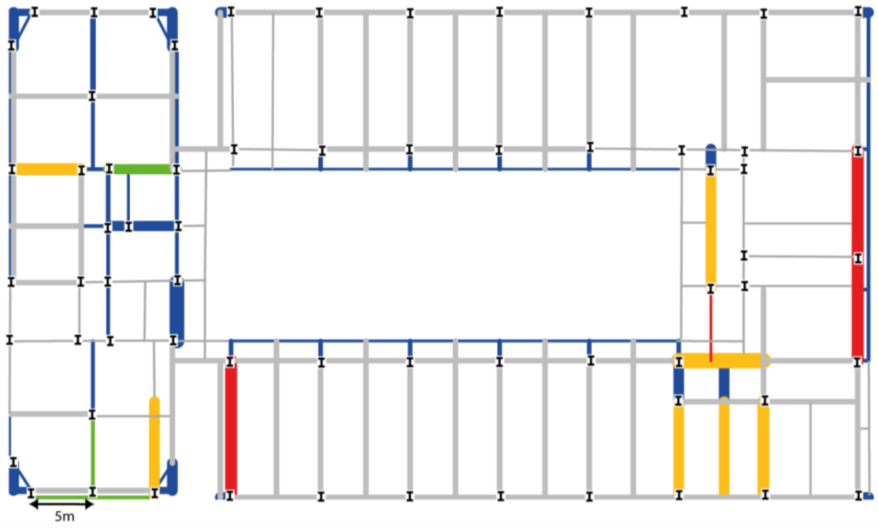

Figure 57: plot of floor showing beams coloured according to utilisation ratio (as per legend pg. S2)

6th floor

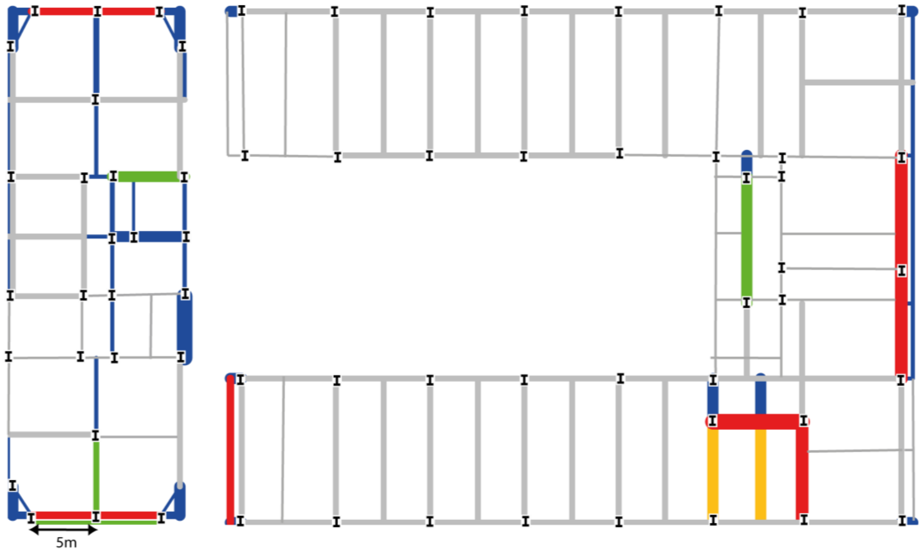

Figure 58: plot of floor showing beams coloured according to utilisation ratio (as per legend pg. S2)

7th – 11th floors

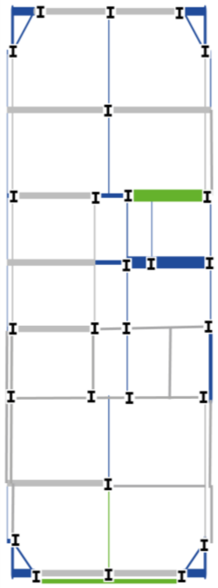

Figure 59: plot of floor showing beams coloured according to utilisation ratio (as per legend pg. S2)

Roof

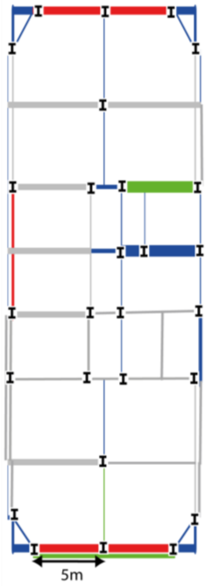

Figure 60: plot of floor showing beams coloured according to utilisation ratio (as per legend pg. S2)

Engineer's comments

Complex procurement involved fabricator twice 'transposing' sections between UK, Russian and Chinese steel catalogues (for cost reasons), choosing heavier section each time 'to be conservative'. Design was originally stress-governed however.

Columns

68 of 215 columns analysed (32%)

Average U/R: 0.62

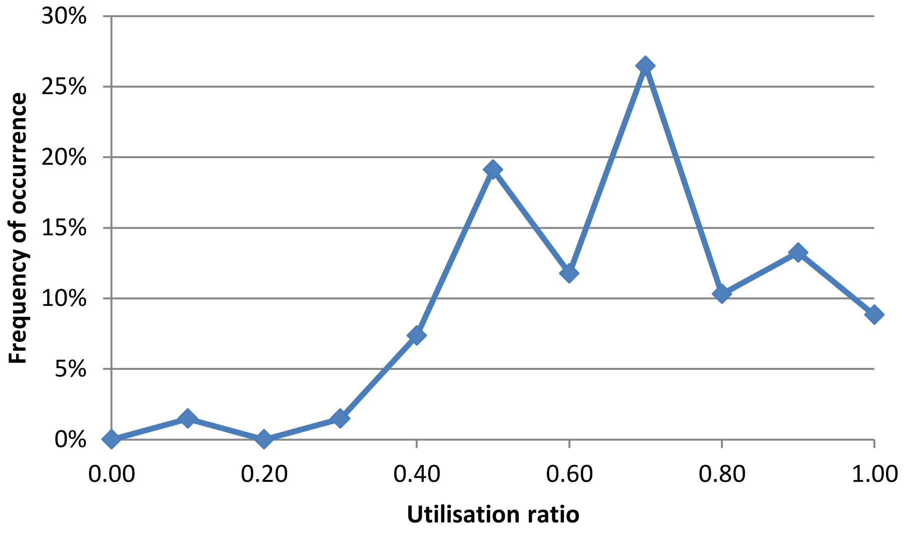

Figure 61: graph of frequency of occurrence against utilisation ratio for columns by floor and overall for building #15

Building #16

Type: mixed use residential

364 of 536 beams analysed (68%)

Table 16: summary of results by floor for building #16

Level	No. beams analysed	% of total steel mass	Avg. U/R	Weighted avg. U/R	Top 5 Beams No.	Top 5 Beams %
Roof	30	12%	0.33	0.51	27	90%
5th floor	72	19%	0.23	0.49	66	92%
4th floor	72	19%	0.22	0.47	66	92%
3rd floor	73	20%	0.24	0.49	67	92%
2nd floor	67	15%	0.19	0.43	61	91%
1st floor	50	16%	0.23	0.39	47	94%
**TOTAL**	**364**	**100%**	**0.23**	**0.46**	**345**	**95%**

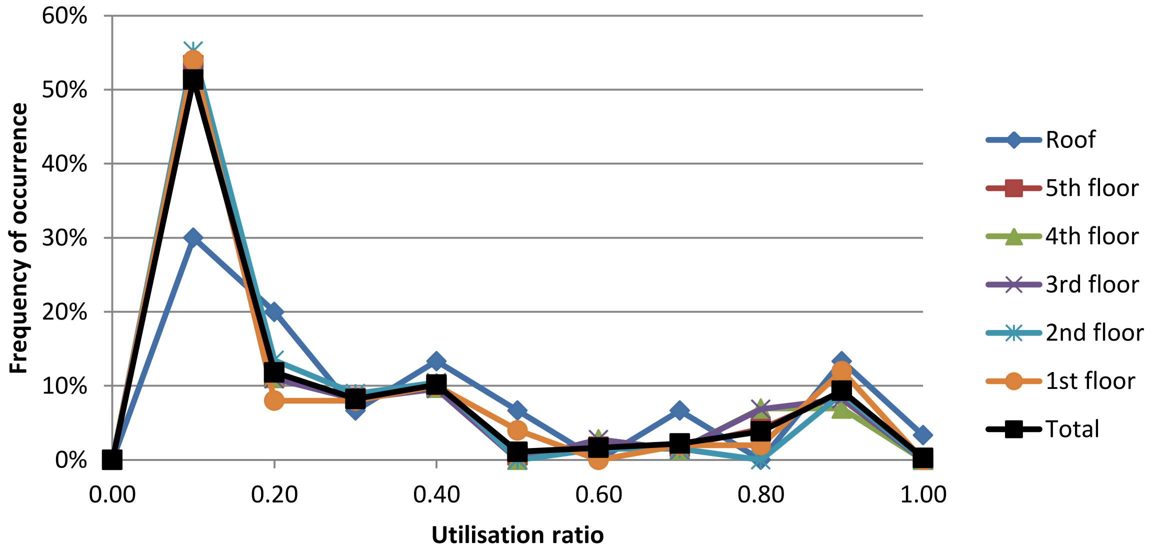

Figure 62: graph of frequency of occurrence against utilisation ratio for beams by floor and overall for building #15

Insufficient data on beam layout were available for this building to create layout plots.

Engineer's comments

Complex procurement involved fabricator twice ‘transposing’ sections between UK, Russian and Chinese steel catalogues, adding weight each time ‘to be conservative’.

Columns

61 of 215 columns analysed (28%)

Average U/R: 0.57

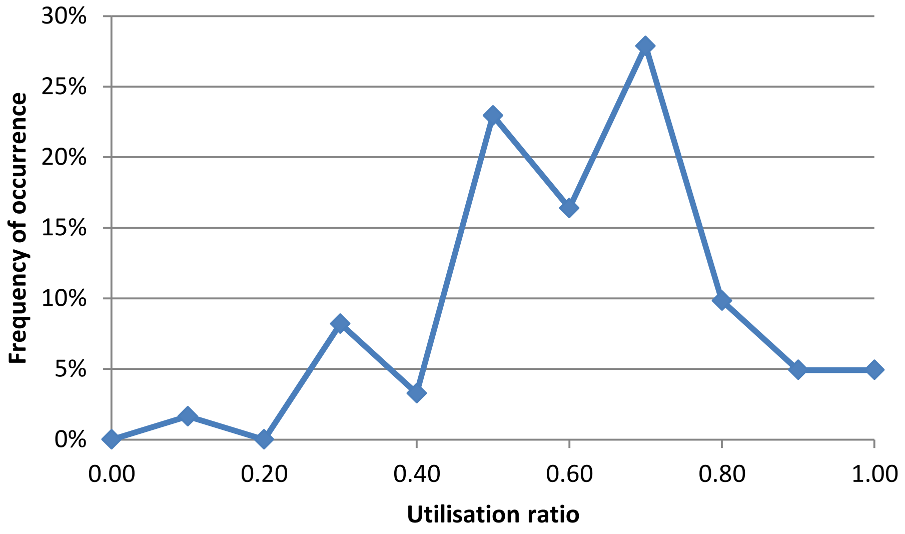

Figure 63: graph of frequency of occurrence against utilisation ratio for columns by floor and overall for building #16

Building #17

Type: mixed-use

631 of 947 beams analysed (67%)

Table 17: summary of results by floor for building #17

Level	No. beams analysed	% of total steel mass	Avg. U/R	Weighted avg. U/R	Top 5 Beams No.	Top 5 Beams %
8th floor	69	10%	0.50	0.68	52	75%
7th floor	67	10%	0.54	0.68	52	78%
6th floor	67	10%	0.54	0.68	52	78%
5th floor	67	10%	0.54	0.68	52	78%
4th floor	67	10%	0.54	0.68	52	78%
3rd floor	67	10%	0.54	0.68	52	78%
2nd floor	105	26%	0.65	0.77	84	80%
1st floor	67	10%	0.50	0.63	50	75%
Others	*55*	*4%*	*0.19*	*0.25*	-	-
**TOTALS**	**631**	**100%**	**0.52**	**0.70**	**514**	**81%**

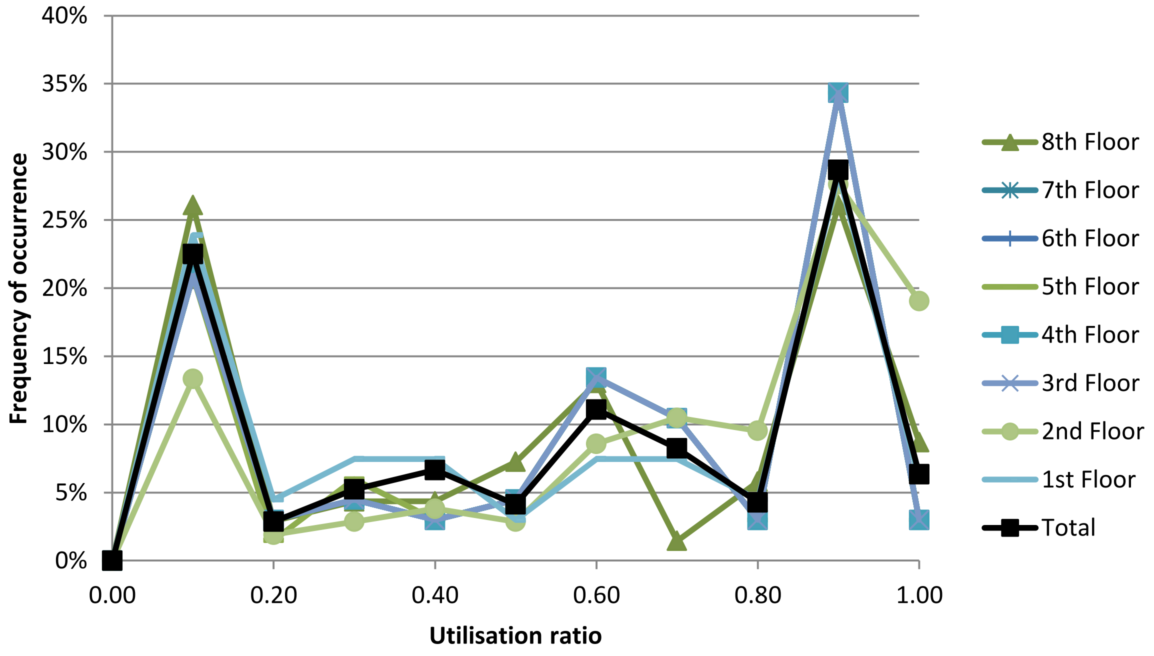

Figure 64: graph of frequency of occurrence against utilisation ratio for beams by floor and overall for building #17

No data on beam layout was available for this building; therefore floor plots could not be created.

Engineer's comments

Vibration governed much of design; this combined with desire to minimise structural depth (to reduce cladding costs) lead to a heavy solution.

Columns

65 of 164 columns analysed (40%)

Average U/R: 0.60

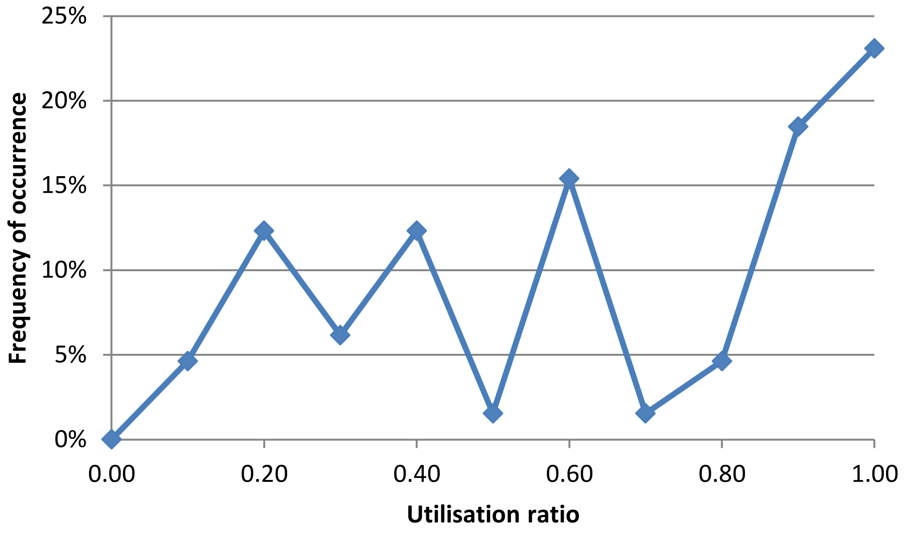

Figure 65: graph of frequency of occurrence against utilisation ratio for columns by floor and overall for building #17

Building #18

Type: office

200 of 316 beams analysed (63%)

Data was only obtained for floors 2 and 10 of this 11-storey building.

Table 18: summary of results by floor for building #18

Level	No. beams analysed	% of total steel mass	Avg. U/R	Weighted avg. U/R	Top 5 Beams No.	%
10th floor	69	36%	0.34	0.57	67	97%
2nd floor	131	64%	0.64	0.71	127	97%
**TOTAL**	**200**	**100%**	**0.54**	**0.66**	**194**	**97%**

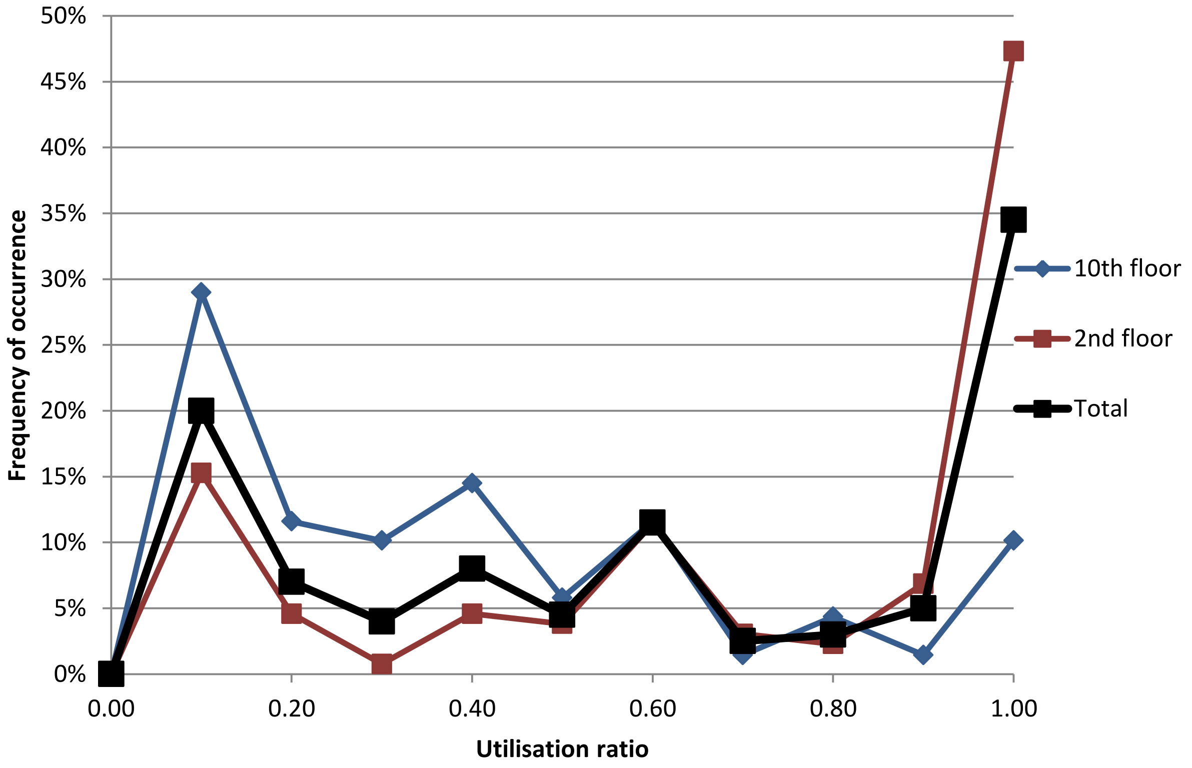

Figure 66: graph of frequency of occurrence against utilisation ratio for beams by floor and overall for building #18

2nd floor

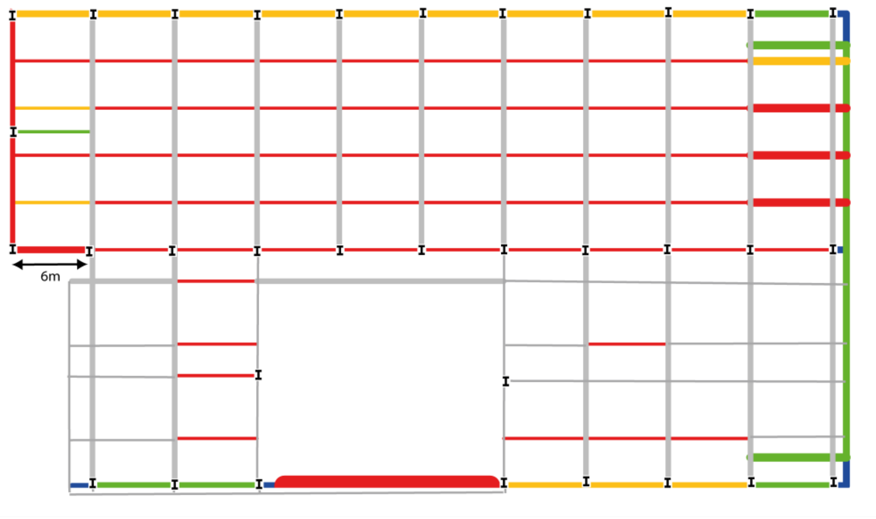

Figure 67: plot of

2nd floor showing beams coloured according to utilisation ratio (as per legend pg. S2)

10th floor

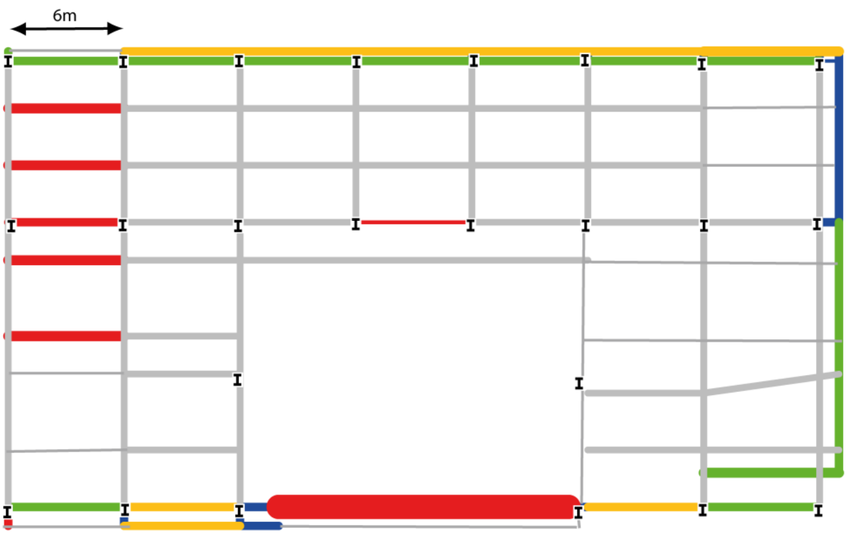

Figure 68: plot of floor showing beams coloured according to utilisation ratio (as per legend pg. S2)

Engineer's comments

Vibration governed much of design. Desire to reduce cladding costs through minimum structural depth sections lead to use of heavy sections.

Columns

57 of 57 columns analysed (100%)

Average U/R: 0.12

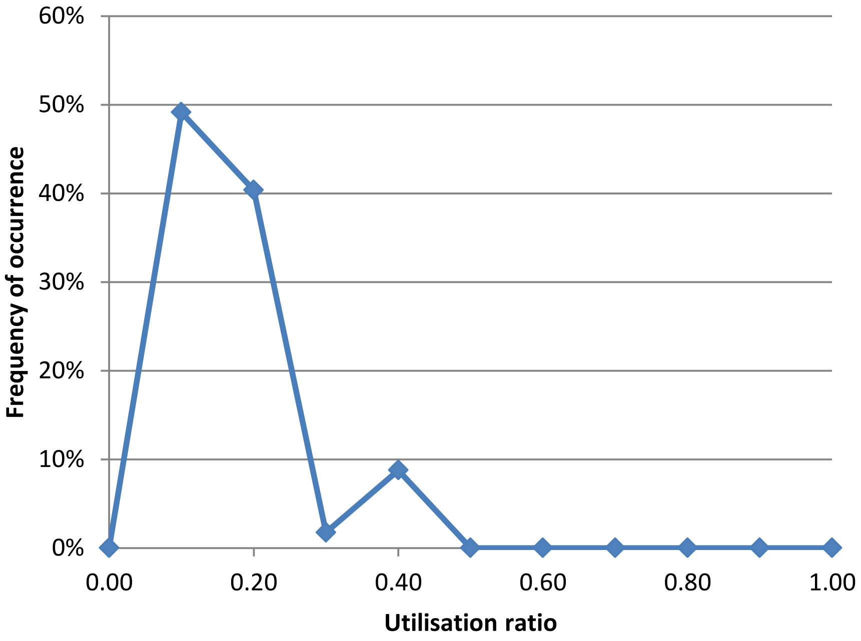

Figure 69: graph of frequency of occurrence against utilisation ratio for columns by floor and overall for building #18

Building #19

Type: school

499 of 527 beams analysed (95%)

Table 19: summary of results by floor for building #19

Level	No. beams analysed	% of total steel mass	Avg. U/R	Weighted avg. U/R	Top 5 Beams No.	%
Roof	30	4%	0.50	0.60	-	-
2nd floor	240	54%	0.30	0.37	176	73%
1st floor	229	42%	0.40	0.48	192	84%
**TOTAL**	**499**	**100%**	**0.36**	**0.43**	355	71%

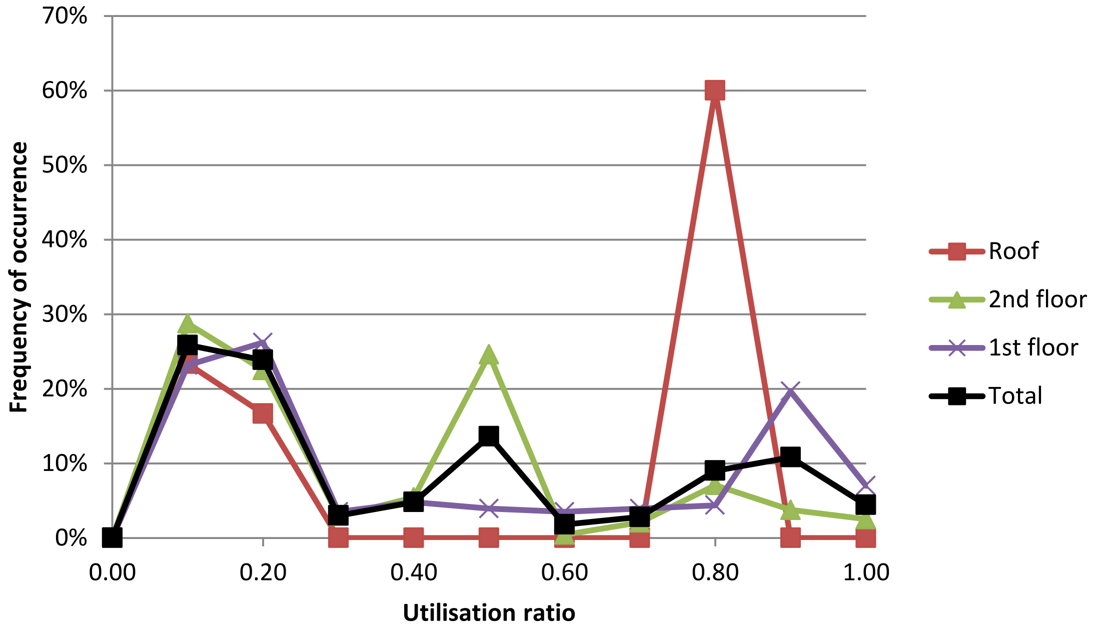

Figure 70: graph of frequency of occurrence against utilisation ratio for beams by floor and overall for building #19

1st floor

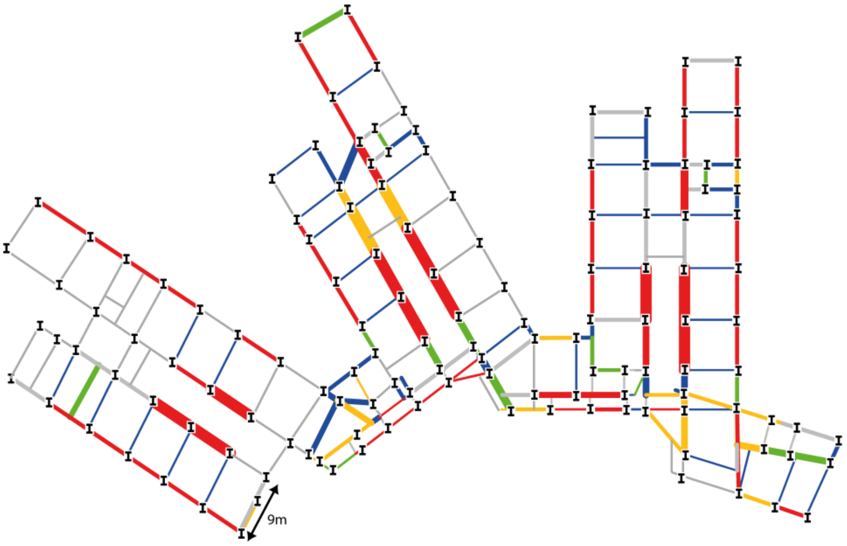

Figure 71: plot of floor showing beams coloured according to utilisation ratio (as per legend pg. S2)

2nd floor

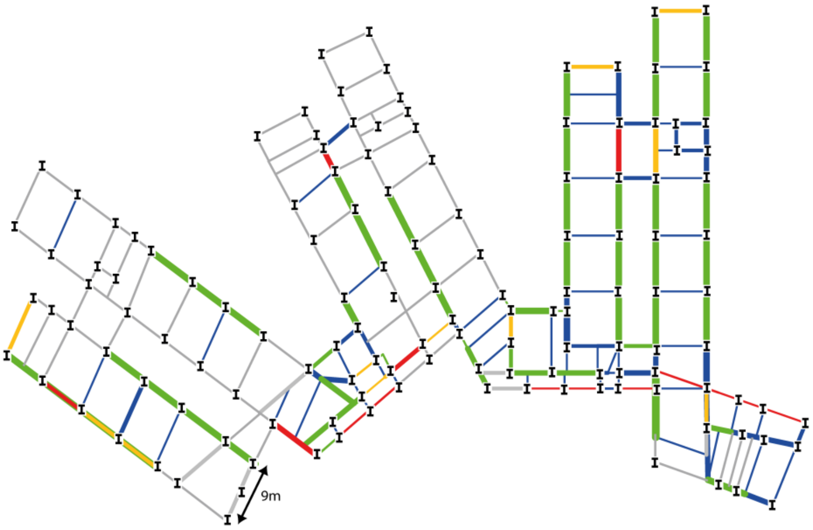

Figure 72: plot of floor showing beams coloured according to utilisation ratio (as per legend pg. S2)

There was insufficient information to construct a plot for the roof level.

Engineer's comments

Primarily deflection governed design.

Columns

150 of 151 columns analysed (99%)

Average U/R: 0.49

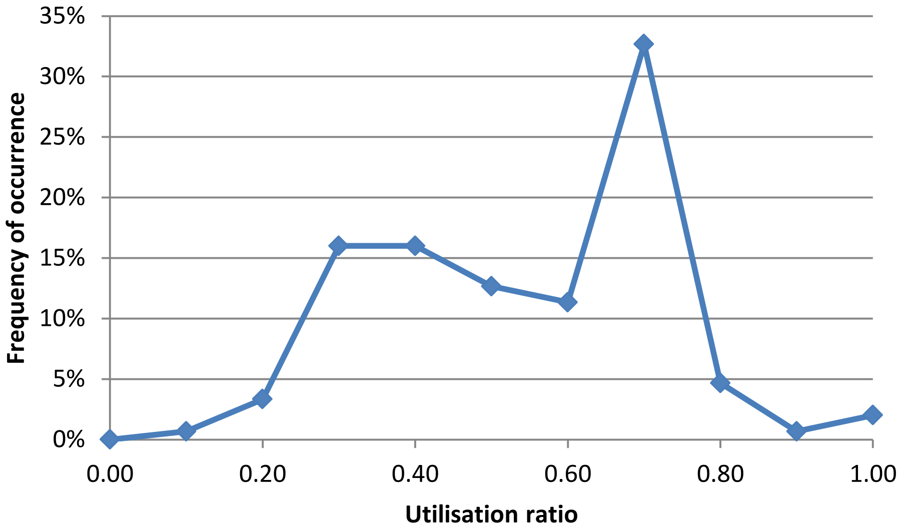

Figure 73: graph of frequency of occurrence against utilisation ratio for columns by floor and overall for building #19

Building #20

Type: school

314 of 322 beams analysed (98%)

Table 20: summary of results by floor for building #20

Level	No. beams analysed	% of total steel mass	Avg. U/R	Weighted avg. U/R	Top 5 Beams No.	Top 5 Beams %
2nd floor	81	30%	0.26	0.54	105	77%
1st floor	119	55%	0.61	0.81	62	52%
Other	*114*	*15%*	*0.09*	*0.35*	-	-
**TOTAL**	**314**	**100%**	**0.33**	**0.66**	**233**	**74%**

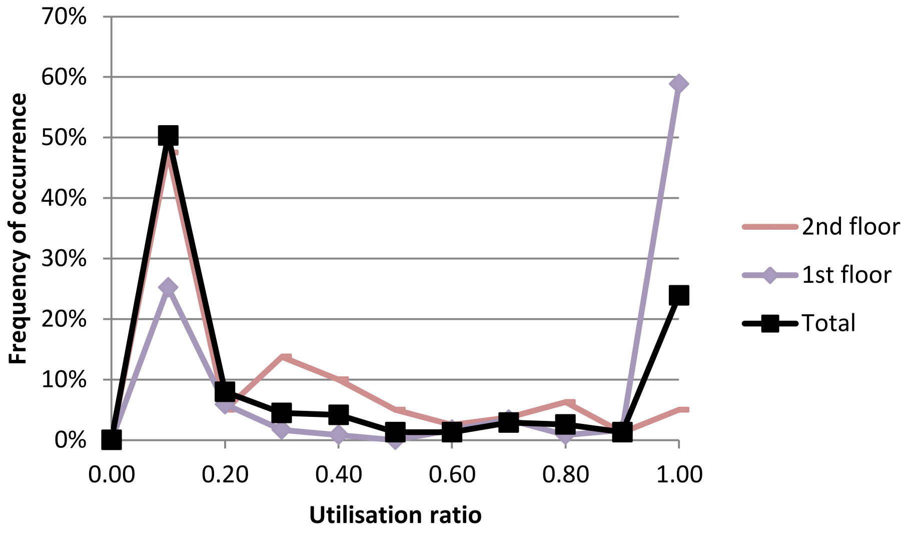

Figure 74: graph of frequency of occurrence against utilisation ratio for beams by floor and overall for building #20

1st floor

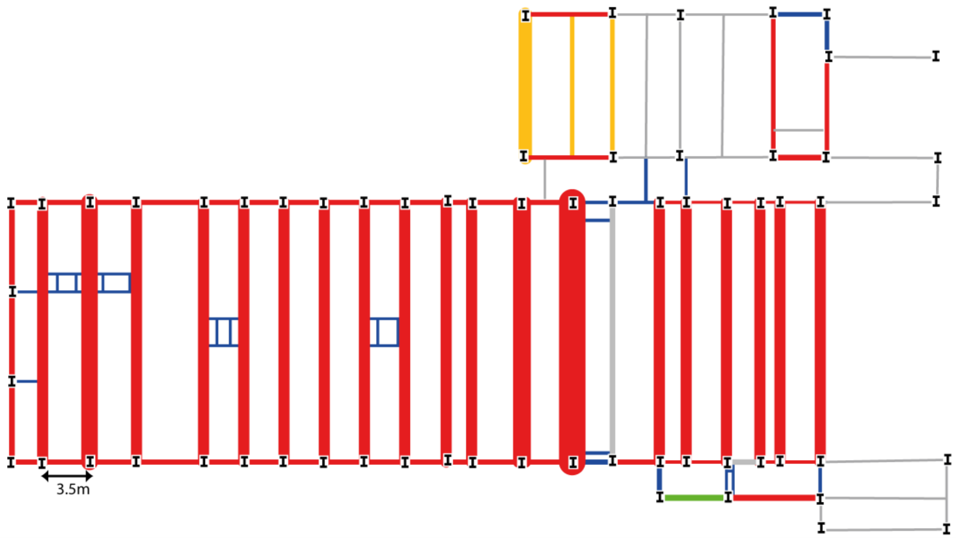

Figure 75: plot of floor showing beams coloured according to utilisation ratio (as per legend pg. S2)

2nd floor

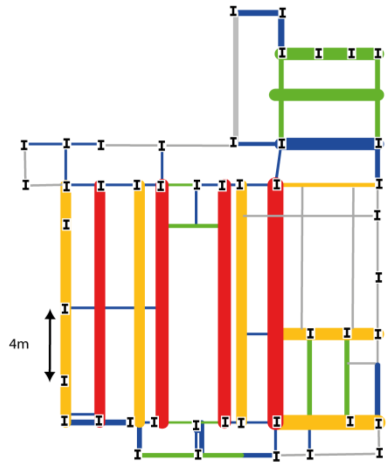

Figure 76: plot of floor showing beams coloured according to utilisation ratio (as per legend pg. S2)

Engineer's comments

Vibration considerations governed design of many areas.

Columns

95 of 96 columns analysed (99%)

Average U/R: 0.35

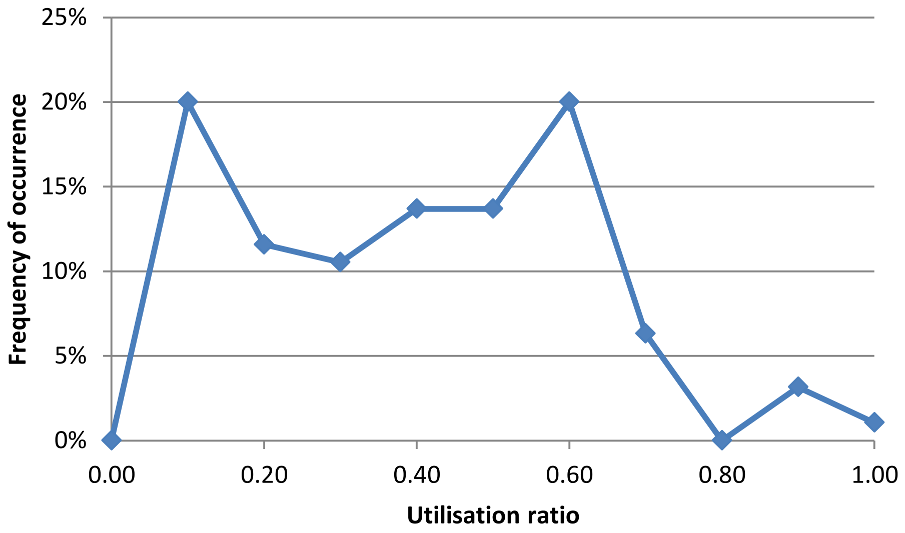

Figure 77: graph of frequency of occurrence against utilisation ratio for columns by floor and overall for building #20

Building #21

Type: residential

71 of 73 beams analysed (97%)

Only data for one floor was available for this building, and no information about beam layout.

Table 21: summary of results by floor for building #21

Level	No. beams analysed	% of total steel mass	Avg. U/R	Weighted avg. U/R	Top 5 Beams No.	Top 5 Beams %
1st floor	71	100%	0.55	0.61	70	99

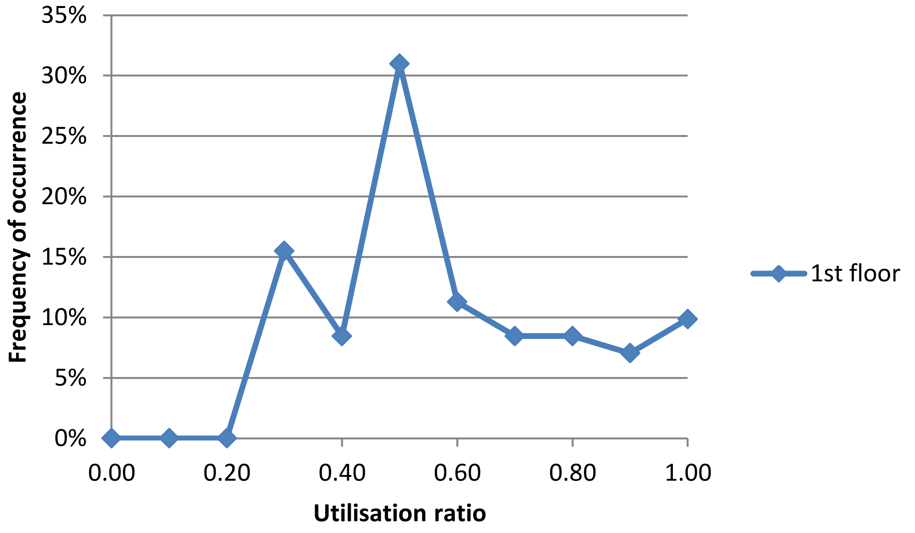

Figure 78: graph of frequency of occurrence against utilisation ratio for beams by floor and overall for building #21

Engineer's comments:

Beam depth specified by architecture – could not even be smaller – therefore very limited range to select from. Vibration governed in some areas.

Columns

213 of 213 columns analysed (100%) – data available for all columns in building

Average U/R: 0.65

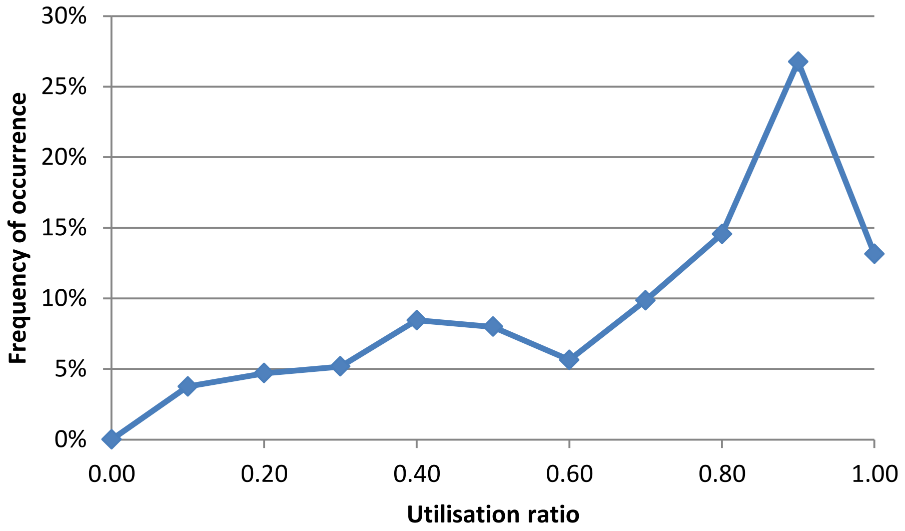

Figure 79: graph of frequency of occurrence against utilisation ratio for columns by floor and overall for building #21

Building #22

Type: school

605 of 613 beams analysed (99%)

Table 22: summary of results by floor for building #22

Level	No. beams analysed	% of total steel mass	Avg. U/R	Weighted avg. U/R	Top 5 Beams No.	Top 5 Beams %
Roof	150	24%	0.36	0.45	119	82%
2nd floor	208	41%	0.57	0.66	174	91%
1st floor	212	33%	0.55	0.64	170	87%
Other	*35*	*2%*	*0.53*	*0.38*	-	-
**TOTALS**	**605**	**100%**	**0.47**	**0.63**	**471**	**78%**

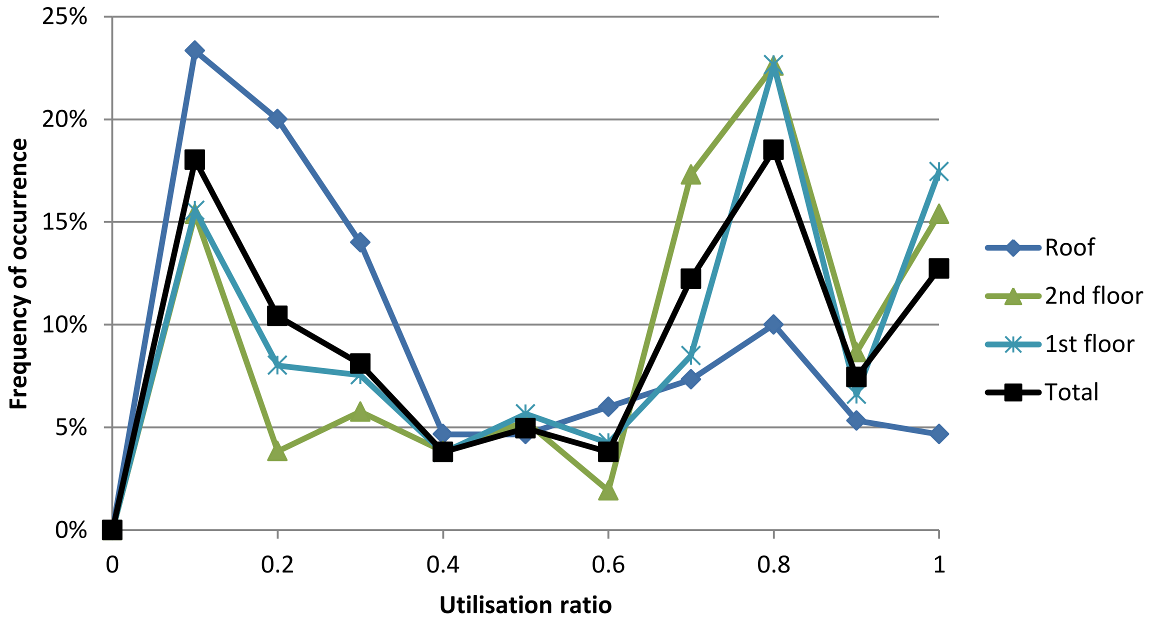

Figure 80: graph of frequency of occurrence against utilisation ratio for beams by floor and overall for building #22

1st floor

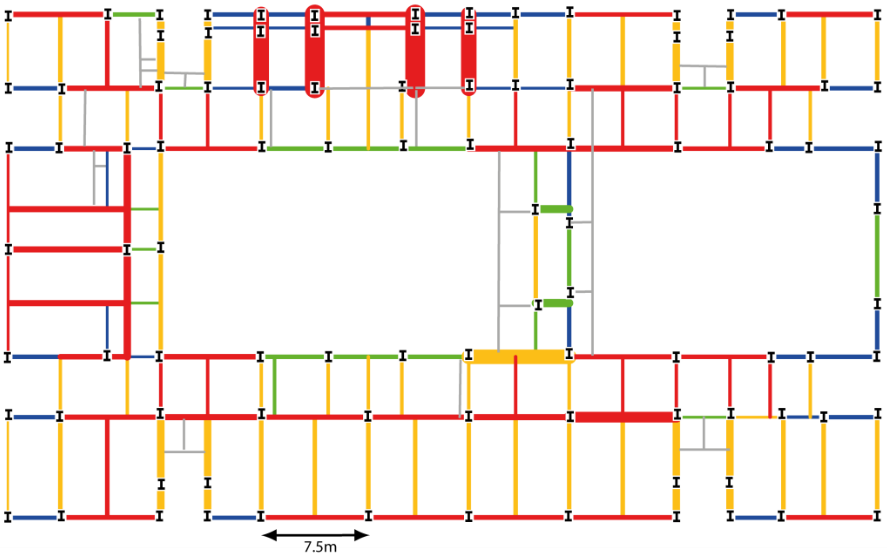

Figure 81: plot of floor showing beams coloured according to utilisation ratio (as per legend 1, pg. 1)

2nd floor

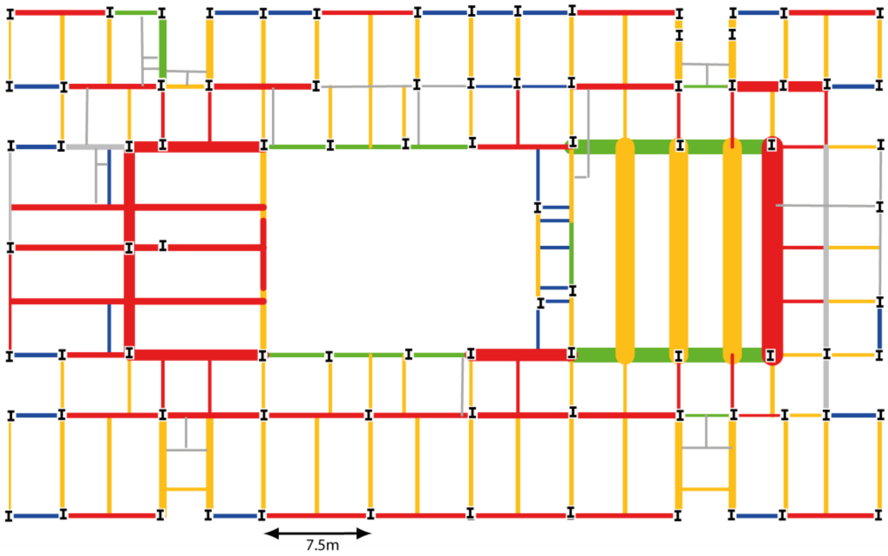

Figure 82: plot of floor showing beams coloured according to utilisation ratio (as per legend 1, pg. 1)

Roof

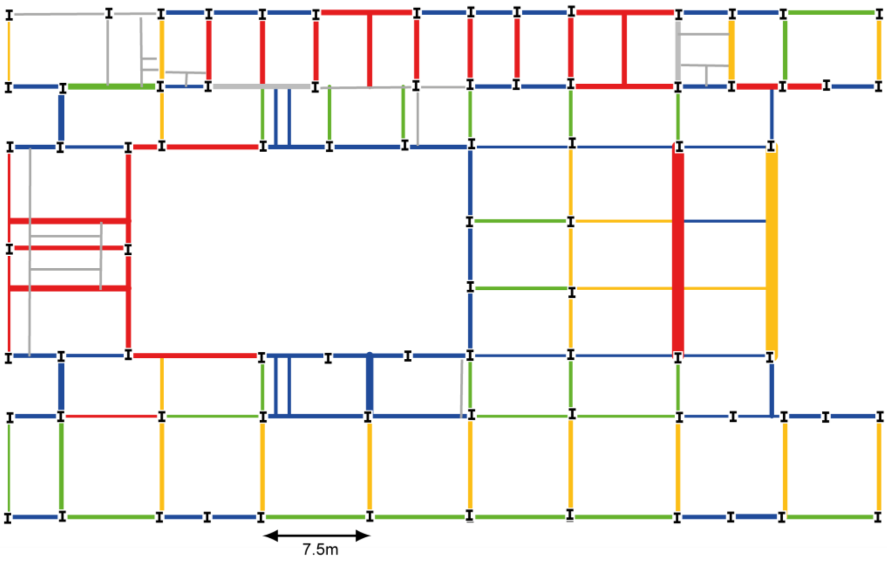

Figure 83: plot of floor showing beams coloured according to utilisation ratio (as per legend 1, pg. 1)

Engineer's comments

Smaller beams oversized to allow faster assembly. Repetition in section sizes encouraged to facilitate faster construction.

Columns

111 of 118 columns analysed (94%)
Average U/R: 0.55

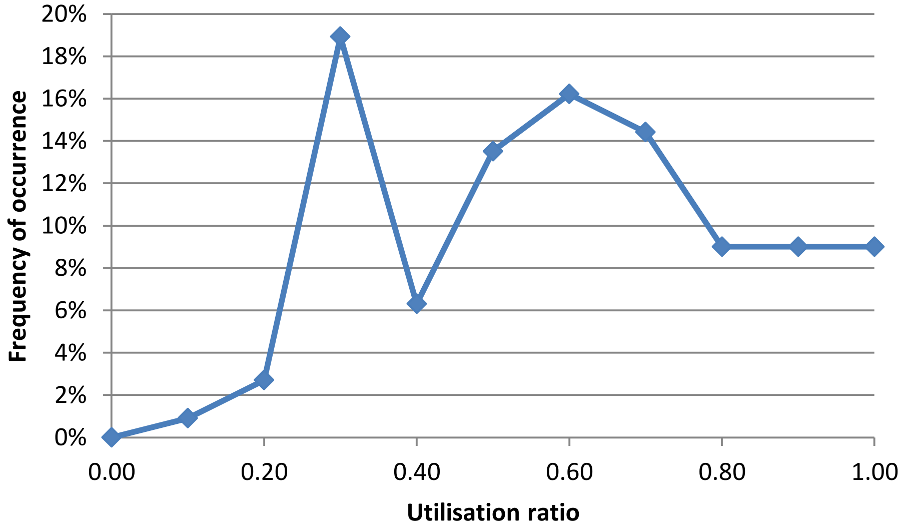

Figure 84: graph of frequency of occurrence against utilisation ratio for columns by floor and overall for building #22

Building #23

Type: school

528 of 558 beams analysed (95%)

Table 23: summary of results by floor for building #23

Level	No. beams analysed	% of total steel mass	Avg. U/R	Weighted avg. U/R	Top 5 Beams No.	Top 5 Beams %
Roof	199	36%	0.34	0.47	173	87%
1st floor	209	46%	0.49	0.66	174	83%
Other	*124*	*18%*	*0.10*	*0.16*	-	-
**TOTAL**	**532**	**100%**	**0.35**	**0.50**	**464**	**87%**

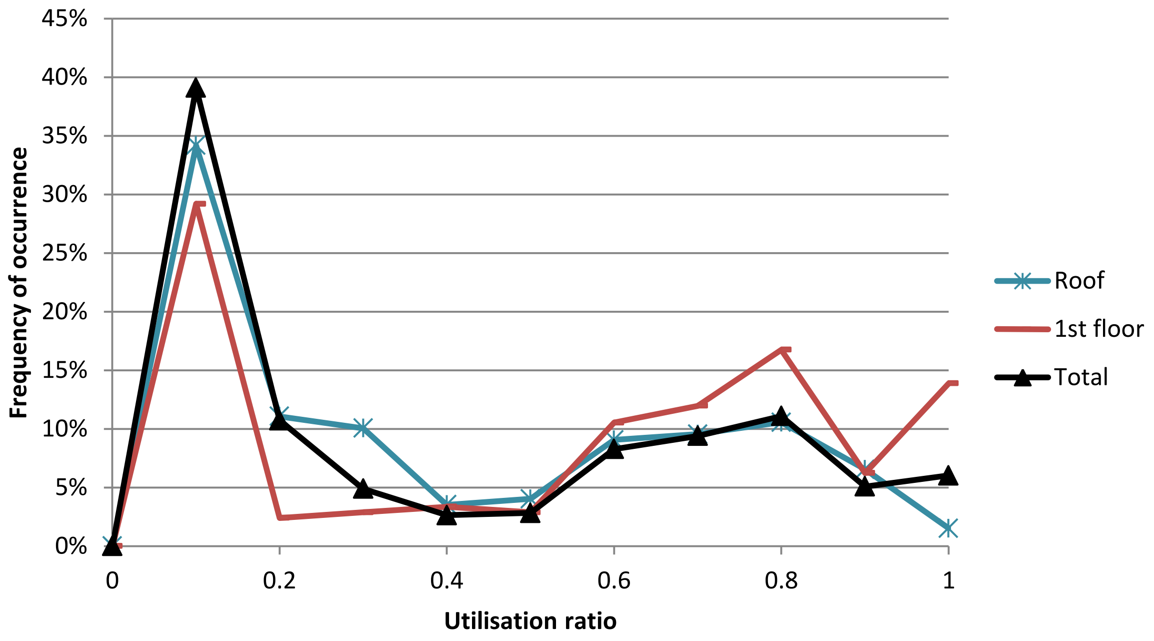

Figure 85: graph of frequency of occurrence against utilisation ratio for beams by floor and overall for building #23

The presence of gridlines with identical names but non-identical coordinates required that the figures below were assembled manually in places, using engineering intuition to assess where beams were located.

1st floor

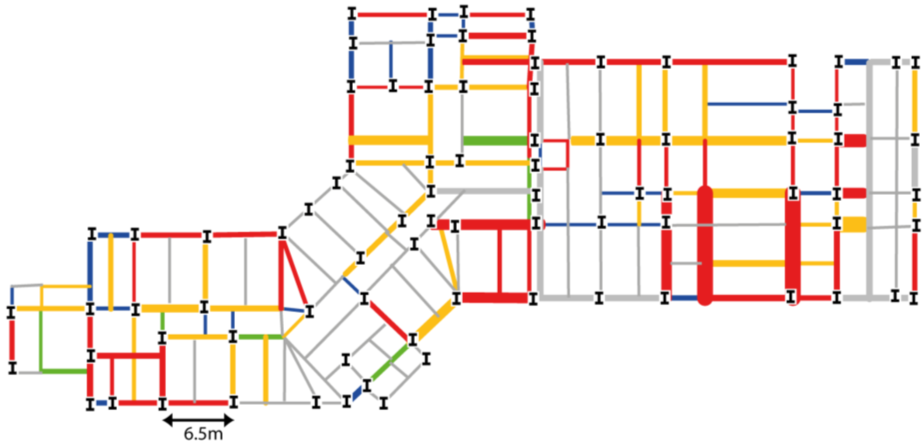

Figure 86: plot of floor showing beams coloured according to utilisation ratio (as per legend pg. S2)

Roof

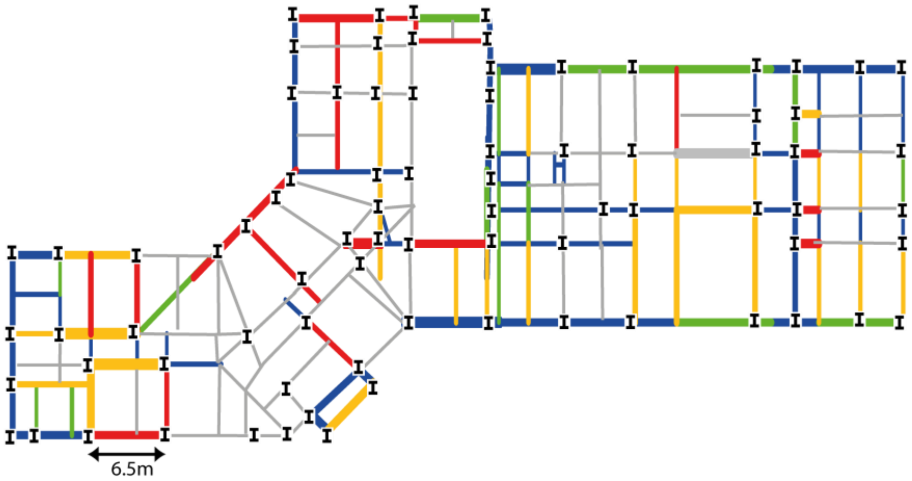

Figure 87: plot of floor showing beams coloured according to utilisation ratio (as per legend pg. S2)

Engineer’s comment

Beams in SE portion governed by vibration, beams along SW governed by stability concerns. Repetition of section sizes encouraged to facilitate faster construction.

Columns

98 of 98 columns analysed (100%)

Average U/R: 0.60

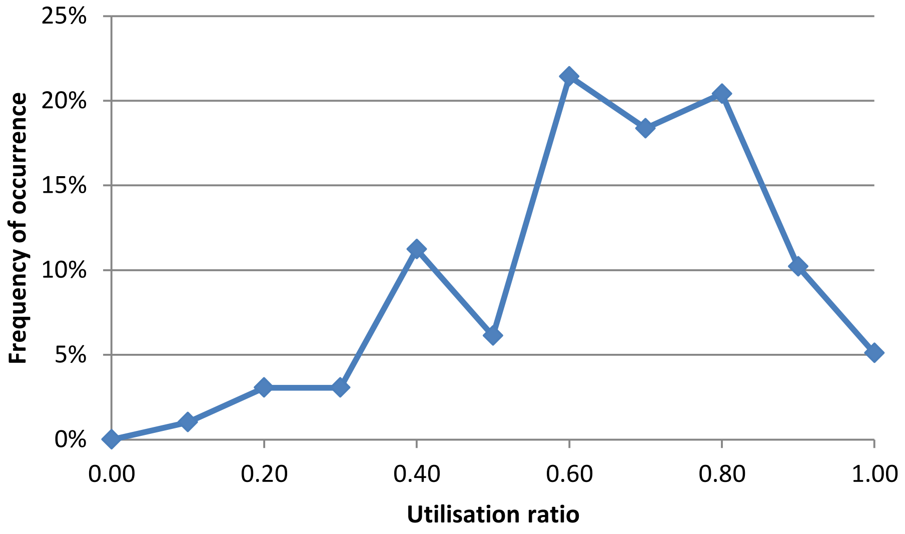

Figure 88: graph of frequency of occurrence against utilisation ratio for columns by floor and overall for building #23

SECTION 2: DESIGN CRITERIA

This section contains details of the design criteria used to evaluate the governing utilisation ratio for each beam and column in each building.

1. Moment capacity
   - About major axis
   - About minor axis
   - Reduced moment capacity – e.g. at holes, near support
     -About major axis
     -About minor axis
2. Shear capacity
   - In direction of minor axis
   - In direction of major axis
3. Axial capacity
4. Buckling*
   - Lateral torsional buckling
   - Strut buckling at various sections
5. Combined axial and moment buckling
   - About major axis
   - About minor axis
6. Deflection
   - Due to dead load
   - Due to imposed load
   - Due to all loads

Other criteria, such as torsion and combined shear and torsion, were included in U/R calculation when specified as governing by calculations or by designer, but otherwise were omitted.

All checks done to worst loading scenario.

*Shear web buckling was checked on a pass/fail basis – i.e. not used to calculated U/R.

SECTION 3: LIST OF QUESTIONS FOR INTERVIEWS

The below template was used when interviewing design engineers. Further unscripted questions were asked to gain more information as necessary.

Utilisation study: questions for interviews

Interviewee: Building name (#):

Email: Emailed in advance on: / /

1. What do you think the average U/R for the building is?
2. At what stage was job handed over? I.e. what stage was model/calc at?
    a. Was model handed to fabricator? Any idea if tonnage up or down for construction?
3. This study included moment, shear, axial, buckling, combined cases and deflection;
    a. Are there any other design cases that governed? If so where?
        i. Vibration
        ii. Construction loads
        iii. Section depth – if so how onerous?
        iv. Connections
    b. Were any design limits more onerous than included in model?
        i. deflection limits
4. How was robustness (disproportionate collapse) accounted for in design and how do you think this would impact on utilisation?
    a. Were any beam sizes changed for robustness specifically?
5. Anything else that will impact my study?
